# Elastomeric 3D‐Printed Microenvironments Enable Nanonewton Force Measurements in Healthy and Diseased Human Pluripotent Stem Cell‐Derived Neuroepithelial Cells

**DOI:** 10.1002/adma.74596

**Published:** 2026-08-17

**Authors:** Pieter F. J. van Altena, Lucia Castillo Ransanz, Ruben H. Guis, Giulia Bergamaschi, Nanne J. Paauw, Urs Staufer, Vivi M. Heine, Angelo Accardo

**Affiliations:** ^1^ Department of Precision and Microsystems Engineering Faculty of Mechanical Engineering Delft University of Technology Delft The Netherlands; ^2^ Department of Child and Adolescence Psychiatry Emma Center for Personalized Medicine Amsterdam UMC Amsterdam The Netherlands; ^3^ Microscopy and Cytometry Core Facility Department of Molecular Cell Biology and Immunology Amsterdam UMC Location Vrije Universiteit Amsterdam Amsterdam The Netherlands; ^4^ Department of Complex Trait Genetics Centre for Neurogenomics and Cognitive Research Amsterdam Neuroscience Vrije Universiteit Amsterdam Amsterdam The Netherlands

**Keywords:** IP‐PDMS, neuroepithelial cell forces, neuromechanobiology, two‐photon polymerization, wet etching

## Abstract

During neural development, cells generate and respond to mechanical forces within their microenvironment while they migrate and differentiate. These forces are central to the development of the human brain, as they are transduced into intracellular signals that regulate key neurobiological processes and may be involved in pathological processes. However, measurement of such forces in the nanonewton (nN) range within a three‐dimensional (3D) microenvironment remains technically challenging and computationally intensive. Here, we present a solution to this challenge, based on elastomeric 3D microstructures fabricated via two‐photon polymerization (2PP) and a tailored wet‐etching process. The resulting free‐standing beam architectures enable the quantification of nN forces exerted by pluripotent stem cell‐derived neuroepithelial progenitor cells. We characterized the morphology of the cells and the forces they exerted using live‐cell confocal imaging microscopy, scanning electron microscopy (SEM), supported by an in‐house routine for intensity‐based deflection measurements with sub‐pixel resolution, and atomic force microscopy (AFM). Using this platform, we precisely quantified neuronal traction forces down to 1.2 nN from both healthy neural cells and those carrying a TSC2 gene mutation linked to tuberous sclerosis complex (TSC). The proposed elastomeric microenvironment paves the way for investigating cytoskeletal mechanics, neuromechanobiology in 3D, and for developing in vitro disease treatment models.

## Graphical abstract

We present elastomeric three‐dimensional (3D) microstructures fabricated via two‐photon polymerization (2PP) and post‐processed through wet etching, for quantifying nanonewton (nN)‐scale forces applied by healthy and diseased neural cells. The mechanically characterized free‐standing beam architectures enable measurement of traction forces of pluripotent stem cell‐derived neuroepithelial progenitors. This platform supports the investigation of cytoskeletal mechanics in 3D for neuromechanobiology and disease modeling.

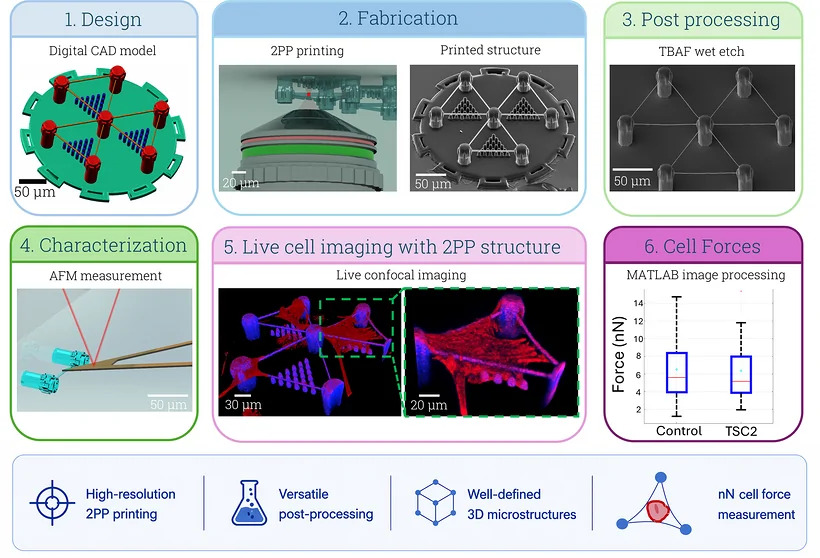

## 1 Introduction

Neural development is governed by a complex interplay of chemical, physical, electrical, and mechanical cues [1, 2]. Over the past decades, substantial progress has been made in understanding how these signals influence neurogenesis and neurodevelopment [2, 3, 4, 5, 6, 7, 8, 9, 10]. While the role of chemical cues in neural development has been extensively investigated, the contribution of mechanical cues, such as extracellular matrix (ECM) stiffness, hydrostatic pressure, fluid shear stress, and tensile forces [11], has received far less attention and remains relatively underexplored [1].

Specifically, tensile forces arise from both cell–cell and cell–ECM interactions during processes, such as migration and differentiation [12, 13]. In the developing brain, these forces play a critical role in regulating the proliferation, migration, and differentiation of neuroepithelial stem cells (NES), affecting neuronal and glial organization and function [2, 14]. Mechanotransduction mechanisms convert these forces into intracellular signals that modulate cell behavior. For instance, mechanosensitive lineage decisions in neural stem cells are influenced by activation of the stretch‐sensitive ion channel Piezo1, highlighting a direct link between mechanical input and developmental fate decisions [15]. Beyond influencing development, mechanical cues also shape neuronal morphology and connectivity, highlighting their central role in brain architecture and function [1].

Disruptions in force generation or mechanotransduction can impair normal brain development [1, 11, 13, 15] and have been implicated in several neurological diseases, including Alzheimer's disease [16], Parkinson's disease [17], and multiple sclerosis [18], where altered tissue mechanics and mechanosensitive signaling pathways contribute to disease progression. Cytoskeletal dysfunction has been suggested to play a role in the onset of Parkinson's disease [19], and altered mechanotransduction was reported in glioblastoma [20]. From a mechanical perspective, research has predominantly focused on neural cell responses to externally applied forces, with fewer studies evaluating forces generated by the neural cells themselves [14].

Noteworthily, neural cells actively probe their mechanical environment even in the absence of externally applied forces, motion, or cues, through intrinsic contractile forces generated by the actomyosin cytoskeleton [13]. The ability of neural cells to exert forces is crucial for key neurodevelopmental processes, such as growth cone‐driven axon extension, neuronal migration [21], and interkinetic nuclear migration [22]. Dysregulation of mechanotransduction pathways has also been implicated in genetic disorders. Among these, tuberous sclerosis complex (TSC) is caused by mutations in either of the TSC genes (TSC1 or TSC2), which regulate mTOR signaling and cytoskeletal disorganization [23]. TSC is characterized by noncancerous tumor formation in the brain and other organs and is often associated with a series of neurological manifestations, including intellectual disability, autism, and seizures. Regulation of the actin cytoskeleton by TSC genes is intricate, as TSC1 and TSC2 act both cooperatively and independently to regulate the actin cytoskeleton through mTOR‐related and other mechanisms. Both human and animal models have shown that TSC mutations alter cytoskeletal dynamics, affecting cell morphology, neurite outgrowth, and migration [24, 25, 26]. It is therefore hypothesized that abnormalities in the cytoskeleton may affect cellular morphology and alter the forces exerted by cells on their microenvironment.

Several methods have been developed to measure traction forces exerted by single cells on their environment, including micropillar arrays [27, 28], traction force microscopy (TFM) [29], atomic force microscopy (AFM) [30], and towing needles [2, 31, 32, 33]. These techniques have provided valuable insights. For example, using 2D TFM, Kumarasinghe et al. showed that the traction force of cortical neurons has a magnitude of 5–11 nN [30]. Shi et al. employed micropillar arrays to measure localized sub‐nanonewton (nN) forces of neural stem cells during differentiation. However, these techniques typically force cells to grow in a 2D or 2.5D environment, affecting the morphology of the cells and causing them to flatten and spread [2], which is not representative of the in vivo three‐dimensional (3D) configuration of brain tissue.

Three‐dimensional TFM partially addresses this limitation by embedding cells in biopolymer matrices such as collagen, fibrin, or Matrigel [34]. Three‐dimensional TFM is a powerful technique that provides physiologically relevant results, but it is experimentally complex and sensitive to variability in terms of hydrogel composition, batch‐to‐batch variability [35], stiffness, swelling, degradation, and pH, which can compromise measurement accuracy and reproducibility [36]. For example, swelling can occur if the hydrogel is not osmotically balanced with the cell culture media or if the gel is pH‐sensitive, thus causing degradation and affecting the results [36].

Recent advances in direct laser writing techniques for microfabrication, such as two‐photon polymerization (2PP), have created new possibilities for fabricating complex 3D microstructures and precisely tuning the mechanical properties of materials [37, 38]. Here, we present a force‐sensitive, elastomeric, reproducible, and mechanically characterized 3D scaffold fabricated by 2PP. The microenvironment is constituted by free‐standing (sub)micrometric beams that enable the measurement of forces in the single‐nN range, exerted by individual live neural cells attached to the beams. Hence, the platform provides a fully 3D, highly elastic environment in which cellular forces are inferred from cell‐induced deformation of engineered microstructures, allowing direct comparison of forces generated by healthy and diseased NES cells. The 3D‐printed micro‐scaffolds were made of a photocrosslinkable version of polydimethylsiloxane (PDMS), called IP‐PDMS [37, 39]. IP‐PDMS features a Young's modulus (YM) that can be tuned in the kPa to MPa range by adapting the fabrication parameters, and which allows the fabrication of large overhangs (up to 70 µm), as previously demonstrated [37]. This remarkably low YM is essential for the beam deflection sensitivity but is also of physiological importance as brain tissue features a YM in the kPa range [40]. To further enhance force sensitivity, we also developed an innovative wet‐etching post‐processing protocol utilizing tetra‐*n*‐butylammonium fluoride hydrate (TBAF) in dimethyl sulfoxide (DMSO) to reduce the cross‐sectional dimensions of IP‐PDMS beams after printing. While TBAF etching has been applied to thermally cured PDMS [41], its use on 2PP‐fabricated microstructures has never been reported. By combining 2PP fabrication with controlled wet etching, we achieved compliant IP‐PDMS beams up to 70 µm in length with a beam height of 1 µm and beam widths down to 420 nm. The microstructures were characterized mechanically, morphologically, and chemically using AFM and nanoindentation, scanning electron microscopy (SEM), confocal imaging, and Raman spectroscopy, respectively.

High‐resolution live confocal imaging, based on time‐lapse z‐stacks, enabled 3D observation and quantification of beam deflection caused by induced pluripotent stem cell (iPSC)‐derived NES cells from healthy controls and patients with TSC. iPSC‐derived NES cells adhered to and bent the force‐sensitive beams, enabling the measurement of cellular forces. We measured a similar mean in‐plane deflection for healthy and TSC2‐mutated cells, respectively, of 2.46 ± 1.27 µm and 2.35 ± 1.33 µm, which is in contrast to our earlier hypothesis that predicted cytoskeletal alterations for TSC2‐mutated cells. The corresponding forces were 6.50 ± 3.41 nN and 6.35 ± 3.34 nN, respectively, indicating no significant difference between the mean forces exerted by NES cells with and without a TSC2 mutation. The proposed 3D‐printed platform, based on a novel 2PP/wet‐etching protocol of the elastomeric biomaterial IP‐PDMS, offers significantly enhanced force sensitivity compared to previous 3D‐printed scaffolds, and enables the investigation of the influence of three‐dimensionality on forces exerted by healthy and diseased NES cells within a 3D‐engineered environment. Our approach therefore paves the way for a series of investigations aiming at elucidating, with nN sensitivity, the mechanical signatures of other healthy and diseased neural cells (e.g., Alzheimer's, Parkinson's, and cancer), thus offering the opportunity to exploit mechanobiology for prospective disease modeling and in vitro treatment screening.

## 2 Results and Discussion

### 2.1 Design and Fabrication of Force‐Sensitive Structures

#### 2.1.1 Two‐Photon Polymerization of the 3D‐Printed Platform

To achieve sensitive detection of forces generated by individual NES cells, we designed an array of double‐clamped, suspended microbeams with maximized compliance and minimal cross‐sectional inertia. Each beam was fabricated with a nominal length of 75 µm and a width of 0.8 µm, printed using a single voxel height of approximately 3.3 µm to reduce structural stiffness. These beams were printed spanning between two pillars, which had a diameter of 20 µm and at a height of 37 µm from a pedestal. The latter was implemented to prevent collapse of the beams during the development. Figure 1 illustrates the general design and printing strategy. By minimizing the cross section (thus moment of inertia) and maximizing the length of the beam, higher bending sensitivities and thus lower spring constants, can be achieved. The pedestal consists of an array of micropillars (each 10 µm in height, 4.5 µm in diameter and with a pitch of 9.5 µm) designed to modulate the mechanical microenvironment experienced by cells through an effective shear modulus rather than the bulk material stiffness. The cells sense a reduced, geometry‐dependent stiffness (effective shear modulus) that originates from the cells' ability to deform individual pillars [38]. This reduction in apparent stiffness promotes cell infiltration and cell growth into the scaffold and facilitates the formation of a 3D niche.

**FIGURE 1:**
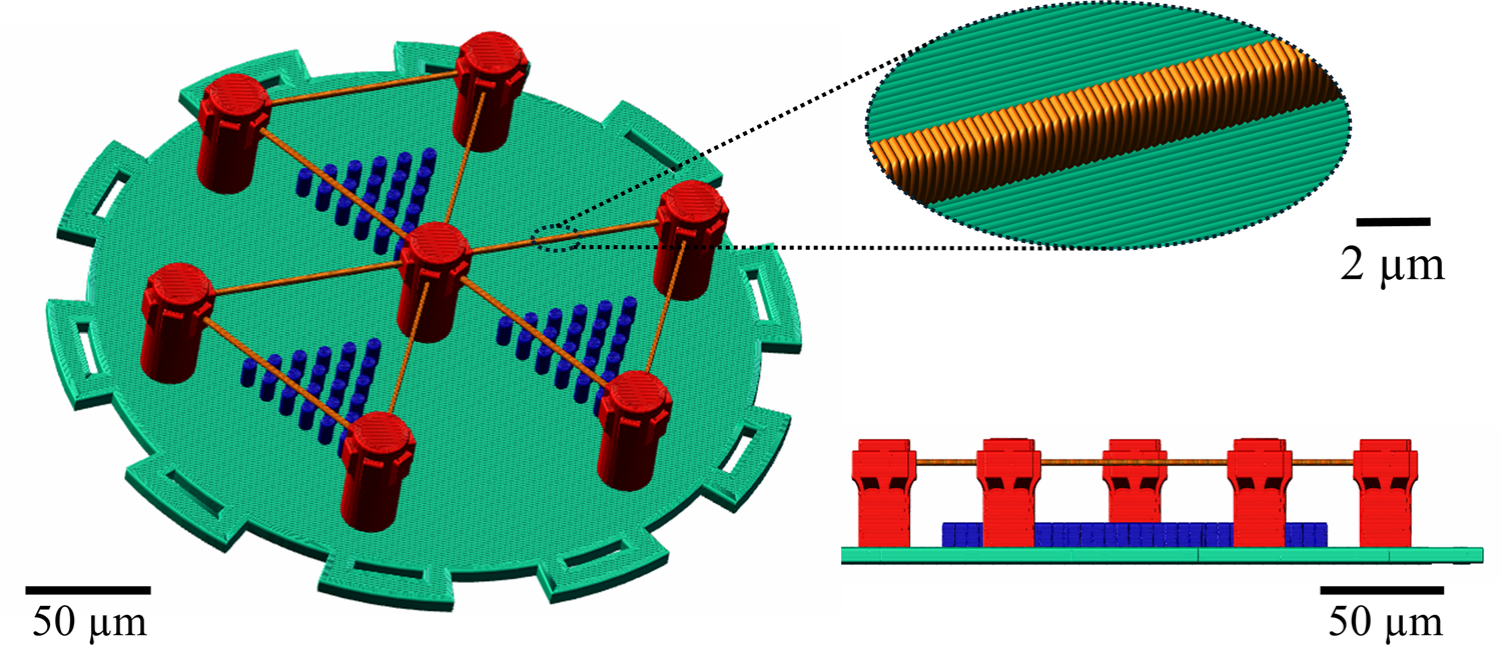
Rendering of the design. Green: pedestal with protrusions to the side for improved print adhesion to substrate. Blue: micropillar array for improved cell adhesion and elevating the cells. Red: supporting pillars for the beams. Orange: beam printed with single voxel lines in a perpendicular direction to the longitudinal axis of the beam.

The structures were manufactured using IP‑PDMS (Nanoscribe GmbH & Co. KG, Karlsruhe, Germany), a photocrosslinkable, elastomeric resin uniquely suited for 2PP fabrication of compliant microstructures due to its tunable YM in the kPa to MPa range and its capacity to form long, stable overhangs [37]. Printing was conducted using single voxel‑line trajectories to minimize over‐polymerization in Z, enabling the generation of uniform beams across each sample. By printing single slices using hatch lines oriented perpendicular to the longitudinal axis of the double‐clamped beams, narrow and thin beams with unsupported spans were achieved. This fabrication strategy required relatively high laser powers (40–50 mW) in combination with low scan speeds (3–5 mm s^−^^1^) to ensure structural integrity, similar to our previous work [37].

The printing sequence started with a pedestal printed on the substrate with three sets of micropillars. Then the central pillar was printed, followed by additional support pillars at the periphery of the pedestal and the beams in between them. A recording of the simulated print and the printing process can be found in Videos S1 and S2. Each substrate contained nine beams to improve yield and cell‑beam interaction probability, and each sample comprised nine 3D platforms, each taking approximately 10 min of fabrication time.

SEM was employed to determine the dimensions of the beams after printing. Figure 2 shows how the beams have an average width of 1.05 ± 0.07 µm (*XY* plane, *n* = 3) and a height of 2.96 ± 0.07 µm (*Z*‐axis, *n* = 3). Thus, over‐polymerization mainly occurs along the *XY* plane, given the nominal width of 0.8 µm and voxel height of 3.3 µm. The length of the beam was approximately 70 µm after printing, indicating a shrinkage in the longitudinal direction of the beam. It is important to note that during printing and development in isopropanol, the beam is curved due to swelling, while during rinsing with NOVEC 7100 and after drying, it is straight (see Figure S1).

**FIGURE 2:**
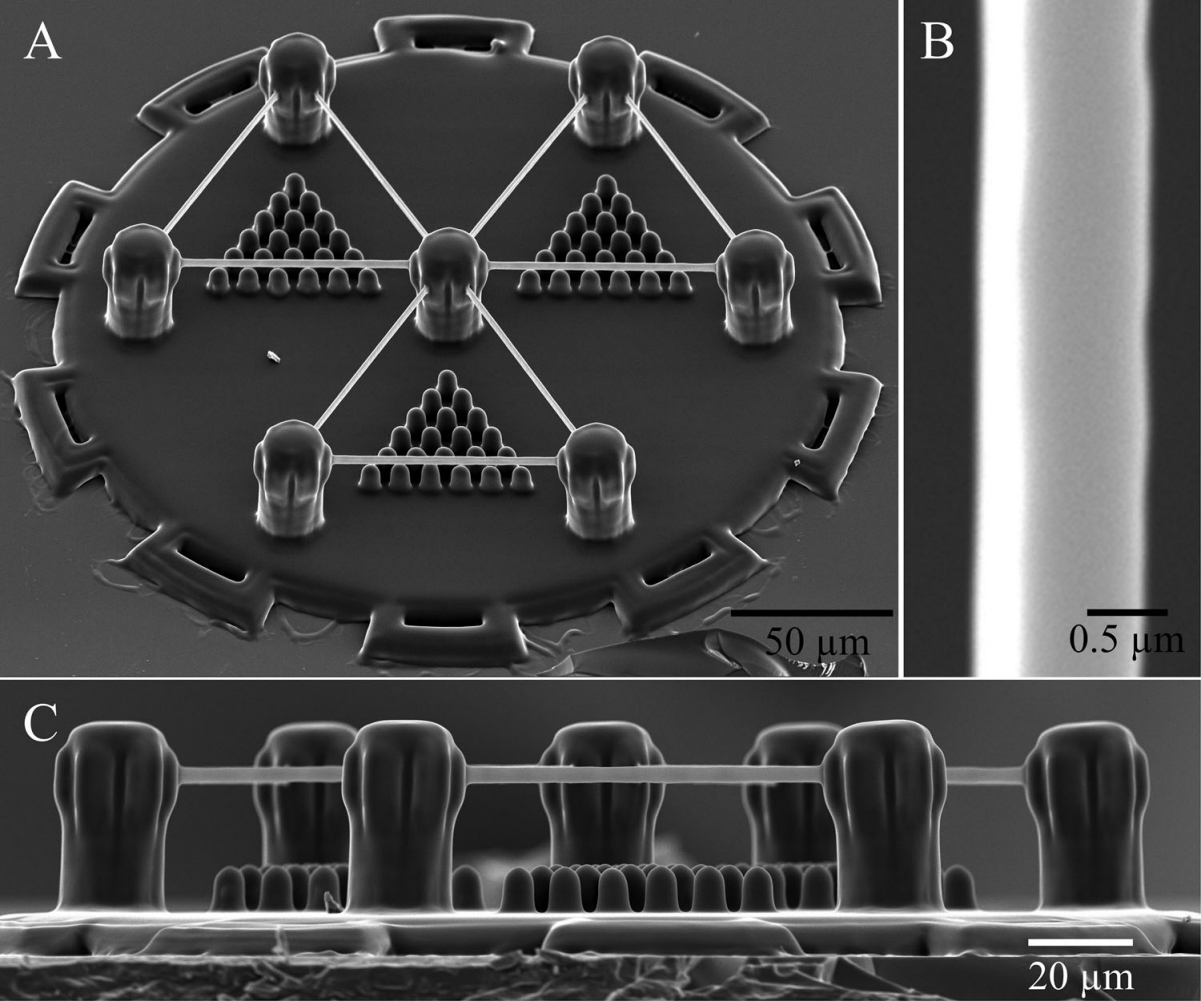
Scanning electron microscopy micrographs of the fabricated IP‐PDMS scaffold. (A) Overview of the full structure showing the circular pedestal with micropillars, supporting pillars, and nine suspended beams arranged at a 45° angle. (B) High‐magnification top‐view image of a representative beam, highlighting its uniform cross‐section. (C) Tilted side view of the structure, illustrating the vertical pillars, suspended beams, and micropillar array on the pedestal.

#### 2.1.2 TBAF Wet Etching

Because the printed features of the IP‑PDMS approach the resolution limits of 2PP for this material, post‑processing by wet etching was developed for further reducing beam dimensions and enhance deflection sensitivity. Tetra‐n‐butylammonium fluoride hydrate (TBAF) in DMSO was selected due to the strong affinity of free fluoride ions for silicon, forming stable Si─F bonds, enabling efficient cleavage of the siloxane (Si─O─Si) backbone within the IP‐PDMS polymer structure. The solvent choice and concentration influence the etch rate and PDMS swelling [41]. DMSO was selected to minimize polymer swelling and provide a low etch rate, thus high controllability.

High‑concentration TBAF (0.88 M) initially revealed that beams can be reduced to widths as small as 432 ± 11 nm and heights of 1017 ± 70 nm (*n* = 3, included in Figure S2). However, such extreme thinning compromised mechanical stability during drying, leading to frequent collapse or detachment (as can be seen in Figure S3).

Figure 3A–C shows structures with 0, 30, and 60 s of etching, respectively, at high TBAF concentration. Figure S4 shows the nonlinear reduction of the dimensions of the beam as a function of etching time. It is important to note that with longer exposure to the etchant, the structural integrity can be affected, resulting in delamination or collapse of the structure, as shown in Figure S3.

**FIGURE 3:**
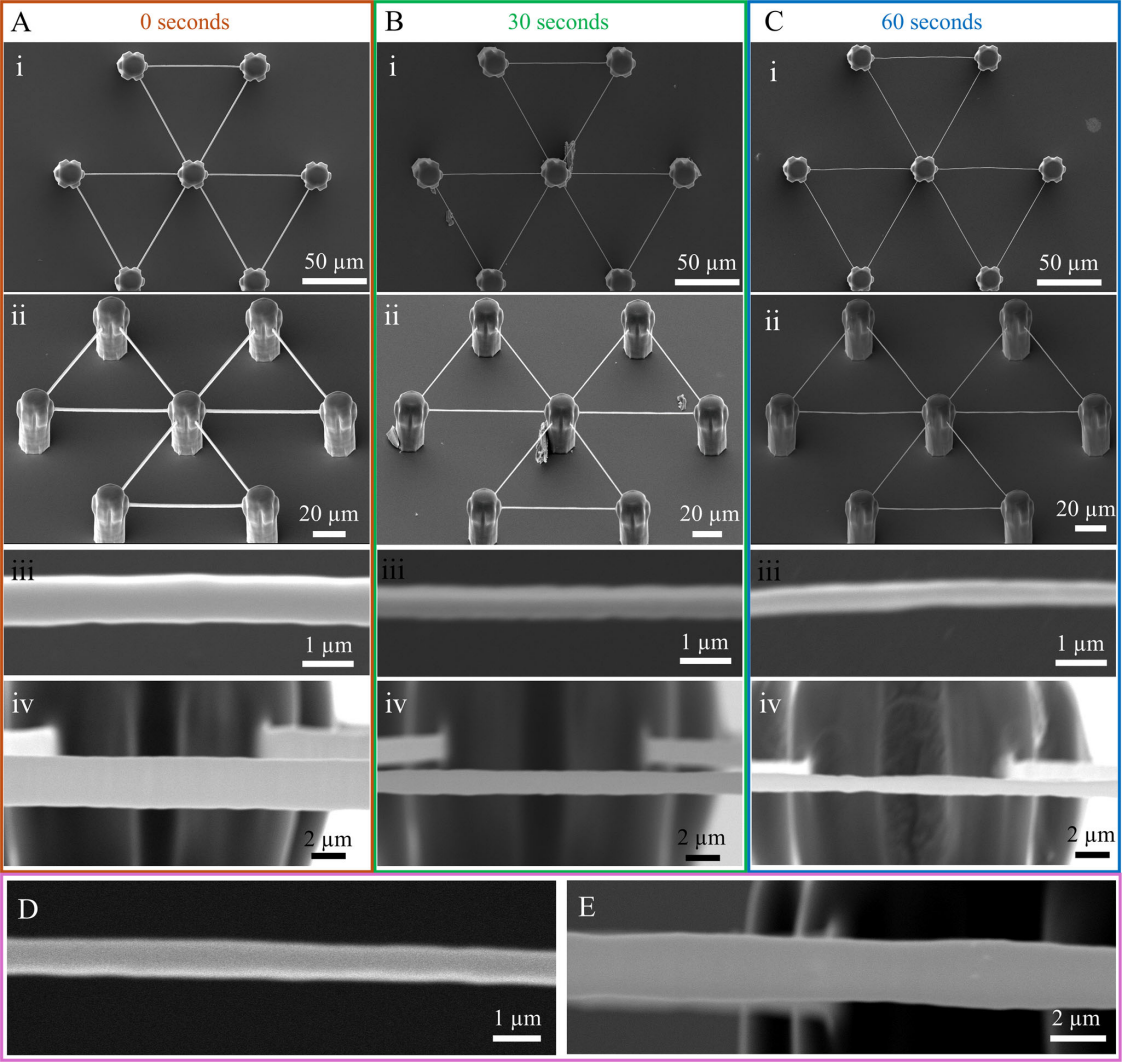
Beam morphology upon different chemical etching times and concentrations. (A–C) High‐concentration (0.88 M) etching at increasing etching times: (A) 0, (B) 30, and (C) 60 s. For each condition: (i) top view of the full structure, (ii) 45° tilted view, (iii) top view of an individual beam, and (iv) side view of the beam highlighting changes in cross‐sectional geometry. Progressive etching results in visible thinning and modification of the beam profile over time (Figure S4). (D) Top view and (E) side view of beams with final etching concentration (0.57 M) employed for live‐cell culture.

To balance sensitivity with structural integrity, a lower concentration of TBAF (0.57 M) was adopted for cell‑experiment samples. At this concentration, the dimensions of the beam were reduced by approximately 400 nm laterally (in the *XY* plane) and 700 nm vertically (along the *Z*‐axis) after 1 min of etching. This directionality may be explained by the energy distribution in the voxel of the laser. The z‐extremal points of the voxel exhibit lower energy compared to its center [42], resulting in a lower degree of polymerization, thus higher susceptibility to the etchant. A 1‑min etching period produced beams with target dimensions of 710 ± 30 nm (*n* = 5) in width (Figure 3D) and ∼2.3 ± 0.05 µm in height (Figure 3E). Careful control of drying conditions, including use of NOVEC 7100 and low‑humidity environments, was necessary to mitigate capillary forces and prevent post‑etch collapse. This controlled etching process significantly increased the compliance of the microbeams while preserving yield and stability, crucial for reliable live‑cell force measurements. For the cell culture experiments, beams with a final height of 2.3 µm and width of 0.71 µm were employed.

Raman spectroscopy also revealed that the TBAF‐based wet‐etching process primarily modifies the geometry of the printed structures by reducing the cross‐sectional dimensions of the beams, while preserving the fundamental chemical structure of the polymer (see Figure S5). Finally, we performed quantitative imaging (QI) AFM measurements on the surfaces of non‐etched and TBAF‐etched IP‐PDMS beams using a super sharp silicon tip (SAA‐HPI‐SS, Bruker, nominal tip radius 1 nm). Surface roughness average (*R*_a_) was quantified over the central region of the beam, as indicated by the white dashed boxes in the AFM height maps (Figure S6). The average *R*_a_ values were 20.37 ± 6.00 nm for the non‐etched beams and 18.64 ± 2.00 nm for the etched beams (*n* = 3 per group), thus indicating that the TBAF etching process does not substantially alter the nanoscale surface topography of the beam surface.

The controlled TBAF post‐processing therefore considerably reduces the cross‐sectional dimensions of the 2PP‐printed IP‐PDMS beams. As the spring constant is strongly dependent on the beam geometry, this dimensional reduction decreases the cantilever spring constant, resulting in larger deflections under a given cellular force and, consequently, enhanced force sensitivity. Unlike previous studies that employed wet etching of conventionally molded PDMS microstructures [43] or dry etching of rigid two‐photon‐polymerized devices [44], our approach combines 2PP of elastomeric IP‐PDMS with controlled chemical post‐processing to tailor the mechanical response of free‐standing beams. Furthermore, the suspended beams are integrated into a 3D micropillar architecture that provides a biomimetic cellular microenvironment, enabling cell attachment, spreading, and migration while simultaneously allowing sensitive quantification of cellular traction forces.

#### 2.1.3 Numerical Analysis and Finite Element Modeling

To predict the mechanical behavior of the fabricated suspended (sub)micrometric beams and guide experimental characterization, numerical analysis was conducted using Euler–Bernoulli beam theory. The maximum deflection for a single load (*P*) in the center of the double‐clamped beam is:

(1) $$\;\delta_{max}=\frac{PL^{3}}{192\;EI}\;$$

With *L* the length of the beam, *E* the Young's modulus of the material, and *I* the cross‐sectional moment of inertia (schematic provided in Figure S7).

Because the beams exhibit a slender geometry with bending‐dominated deformation, the analytical formulation Equation (1) provides a reliable first approximation of their stiffness. The maximum deflection under a point load of 1 nN was computed using standard beam deflection equations, incorporating the measured YM obtained from AFM and nanoindentation experiments to estimate the deflection of the beam when subjected to a 1 nN point force at the center of the beam.

The printed beams, characterized by a length (L) of ∼70 µm after shrinkage, were modeled with an obround cross section consistent with the voxel‐based printing process. The YM of the IP‐PDMS printed beam was measured in air using AFM, resulting in an average YM of 6.1 ± 0.53 MPa (*n* = 713). In addition, interferometer‐based nanoindentation was performed on a pedestal with the same printing parameters, yielding an average YM of 2.9 ± 0.07 MPa (*n* = 25), which is in the same range as the one reported in our previous work [37]. The average YM obtained from AFM measurements was used in the numerical and analytical models, as these measurements were performed locally on the side of an individual printed beam rather than via nanoindentation of the IP‐PDMS pedestal. Figure S8 shows the nanoindentation and AFM results.

Although the YM obtained by AFM (6.1 ± 0.53 MPa) was higher than that measured by interferometer‐based nanoindentation (2.9 ± 0.07 MPa), the AFM‐derived value was used for the force calculations. The difference is attributed to the distinct indentation geometries and probing depths of the two techniques, with AFM employing a relatively sharp 10 nm‐radius tip that primarily probes the near‐surface region, whereas nanoindentation uses a 20 µm‐radius tip with a larger contact area and deeper indentation. Moreover, nanoindentation measurements were performed on a printed pedestal due to geometric constraints, while AFM measurements were carried out directly on the sensing beams. Consequently, the AFM‐derived modulus was considered the most representative mechanical parameter for modeling the cantilever response. As this higher modulus yields larger calculated forces for a given beam deflection, it also provides a conservative upper‐bound estimate of the cellular traction forces.

The moment of inertia was calculated using Equation (2) for obround geometries [45].

(2) $$I_{y}=\frac{b^{3}h}{12}+\frac{\pi b^{4}}{64}$$

with *H* being the total height of the beam, *b* the width of the beam, and *h* = *H*‐*b*. The spring constant, perpendicular to the beam and parallel to the pedestal, that is in the direction of lowest stiffness, can be calculated using the following equation:

(3) $$\;k_{x}=\frac{192EI_{y}}{L^{3}}$$

Calculation of the moment of inertia for this geometry enabled an estimation of the beam's spring constant in the direction of minimal stiffness, yielding a theoretical value of 2.8*10^−4^ Nm^−1^.

These numerical results predict the expected force sensitivity of the system and provide a benchmark for subsequent finite element simulations as well as AFM‑based stiffness measurements.

Finite element modeling (FEM, Figure S9) was employed to further verify the mechanical behavior of the printed structures. The simulations were performed in COMSOL using the structural mechanics module, enabling geometric nonlinearity to account for large deflections. A lateral point force was applied in steps of 0.05 nN. The etched beam was used to simulate the deformation when 1 nN was applied to the center of the beam. The material was modeled as a linear elastic solid using the maximum experimentally measured YM, and a Poisson's ratio of 0.49 [39, 46, 47] was assumed. The full 3D geometry of the beam was meshed without symmetry assumptions, employing a highly detailed mesh to resolve stress concentrations. These boundary conditions enabled accurate prediction of the bending behavior and direct comparison with AFM‑based stiffness measurements performed during experimental characterization in Figure 4. The simulations featured good convergence (<10^−10^) and a final deformation of 1.26 µm under 1 nN load, as reported in Figure S9.

**FIGURE 4:**
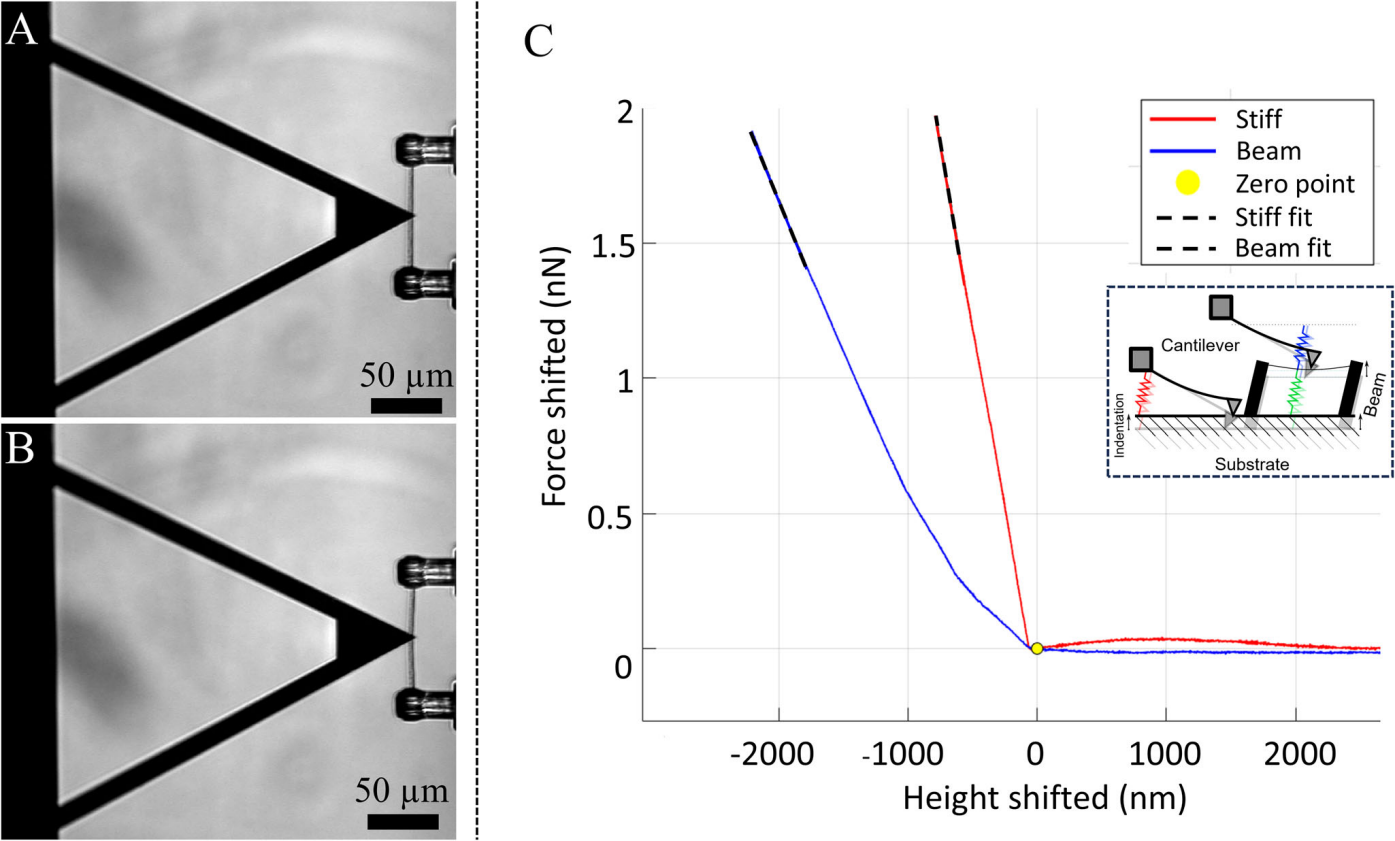
Atomic force microscopy (AFM)‐based mechanical characterization of IP‐PDMS beams. (A) Representative optical image showing the AFM probe positioned in contact with a suspended beam. Force measurements were performed on the lateral side of the beam. (B) Optical image illustrating beam deformation upon vertical loading by the AFM cantilever. (C) Force–displacement curves obtained from measurements on the stiff ITO substrate (red) and the suspended beam (blue) after 53 h in 37°C physiological imaging medium. The curves were aligned in MATLAB JPK processing software, and the linear regions were fitted (dashed lines) to extract the effective stiffness. The intersection point (yellow marker) indicates the zero‐force reference. The inset shows the ideal spring model for the AFM experiment.

Hooke's law Equation (4) was used to calculate the spring constant of the beam.

(4) $$k=\frac{F}{\delta }\;$$

The resulting simulated spring constant is 7.9*10^−4^ Nm^−1^. The difference between the numerical (2.8*10^−4^ Nm^−1^) and FEM solution can result from the automatic inclusion of shear, axial stretching, stress redistribution, and geometric nonlinearities in COMSOL. Together, the analytical and numerical results confirmed that the beams possess sufficient compliance to resolve single‑nN forces exerted by NES cells.

### 2.2 Experimental Characterization of the Force‐Sensitive Structures

AFM was employed to mechanically characterize the microscale beam structures. Mechanical characterization of the beam was carried out by acquiring force–displacement curves at the center of the suspended 2PP printed beam. The substrate was mounted at a 90° angle on the AFM to enable force measurements aligned with the primary direction of cellular traction. To determine the spring constant of the beam, the system was modeled as springs in series Equation (5) and shown in Figure 4.

(5) $$\;\frac{1}{k_{eff}}=\frac{1}{k_{cantilever}}+\frac{1}{k_{beam}}$$

The beam spring constant can be extracted using *S_hard_* and *S_combined,_* defined, respectively, as AFM force/indentation slopes measured on the stiff surface (ITO substrate) and on the beam [48]

(6) $$\;k_{beam}=\frac{k_{cantilever}}{\left(S-1\right)cos^{2}\theta }\;with\;S=\frac{S_{hard}}{S_{combined}}$$

where θ is the angle of the mounted AFM probe (10°). The *k*_cantilever_ should be matched to *k*_beam_, such that 0.3 *k*_cantilever_ < *k*_beam_ < 3.0 *k*_cantilever_ [48].

The AFM probe was positioned above the side of the beam using optical microscopy and engaged in contact mode (Figure 4A). During each measurement, the cantilever was driven vertically while recording the deflection signal as a function of piezo displacement. The applied force was calculated from the measured cantilever deflection and the calibrated spring constant. Figure 4B shows the visible deformation of the IP‐PDMS beam. For each beam, two measurements were performed. One measurement on the stiff ITO substrate on which the structures were printed, and one on the suspended beam. Both measurements were aligned and plotted using MATLAB, and the slope of the linear part was fitted as reported in Figure 4C. Equation (6) was used to calculate the stiffness of the beam, resulting in IP‐PDMS beams spring constants of 0.014 and 0.0023 Nm^−1^, for etched beams in air and aqueous environments, respectively. IP‐PDMS exhibits viscoelastic behavior, particularly in hydrated environments. It is noteworthy that while the YM of IP‐PDMS (2.9–6.1 MPa) remains higher than that of native brain tissue (typically 0.1–10 kPa), the bending stiffness of the beams, rather than the bulk material modulus, can influence the mechanical boundary condition experienced by the cells [38]. To evaluate the mechanical stability of the IP‐PDMS beams under prolonged culture conditions, beams were incubated in physiological imaging medium (Invitrogen, A59688DJ) at 37°C and measured by AFM indentation after 1, 24, and 53 h. The measured beam stiffnesses were 0.0022 ± 0.0003 N m^−^^1^, 0.0023 ± 0.0002 N m^−^^1^, and 0.0022 ± 0.0001 N m^−^^1^ (*n* = 9, Figures 4C and S10). No significant change in stiffness was observed, suggesting that the aqueous environment does not influence the spring constant over the time scale of the live‐cell experiments. The observed stiffness retention is consistent with previous studies reporting that hydration‐induced swelling of PDMS‐based materials reaches equilibrium rapidly in aqueous media, limiting further time‐dependent changes in mechanical behavior during extended incubation [49, 50].

Several 3D‐printed force‐sensing platforms have been developed to quantify cell‐generated forces in biomimetic microenvironments. Klein et al. demonstrated a scaffold‐based approach using 3D‐printed beams to measure cardiomyocyte‐generated forces from beam bending observed by phase‐contrast microscopy. They reported an effective spring constant of approximately 50 nN µm^−^^1^ (equivalent to 0.05 N m^−^^1^) [31]. Perera et al. subsequently developed two‐photon‐polymerized microcage structures containing suspended wires for simultaneous confinement of single mesenchymal stromal cells and force measurement, with the most compliant wires exhibiting a spring constant of approximately 16 nN µm^−^^1^ (equivalent to 0.016 N m^−^^1^) [51]. More recently, Ucla et al. reported deformable photopolymerized fiber arrays, in the presence of HUVECs, fibroblasts, and macrophages, with stiffness values ranging from 3.5 to 17.6 nN µm^−^^1^ (equivalent to 0.0035–0.0176 N m^−^^1^) for 80 µm‐long fibers measured using AFM during experiments lasting up to 16 h [52]. In comparison, the wet‐etched IP‐PDMS beams with a length of 70 µm developed in this work for measuring donor‐derived iPSC neural cells exhibit a low effective spring constant of approximately 0.0023 N m^−^^1^ while maintaining structural stability during prolonged live‐cell experiments up to 64 h. This high sensitivity is achieved through the combination of a low YM elastomeric 2PP material and a controlled TBAF‐mediated reduction of the cantilever cross‐section. The resulting force sensitivity is comparable to the most compliant 3D‐printed force‐sensing architectures reported to date, and approaches the lower range commonly reported for micropillar‐based force sensors [53].

By maintaining the structures in a hydrated state after fabrication, avoiding drying‐induced capillary forces, and using higher TBAF concentrations, even lower spring constants (approaching 0.001 N m^−^^1^), and consequently higher force sensitivities, could potentially be achieved. This may further extend the applicability of the platform to the measurement of extremely small cellular forces while preserving the advantages of a 3D biomimetic microenvironment.

### 2.3 Confocal Microscopy of Fixed Cells and SEM Imaging on the 3D Microenvironment

To assess the suitability of the fabricated 3D platform for cell adhesion and growth, an initial set of experiments was performed using confocal fluorescence microscopy on chemically fixed samples and SEM on dehydrated samples. This enabled both qualitative and morphological evaluation of cell behavior on the 3D‐printed microenvironment.

Confocal laser scanning microscopy was performed to visualize cell morphology and distribution within and on top of the 3D architecture. Cells were stained with phalloidin and DAPI to visualize the F‐actin and nuclei, respectively. Z‐stack image series were acquired using a high numerical aperture objective, enabling 3D reconstruction of cell positioning relative to the scaffold features. Particular attention was given to studying  the ability of cells to bridge and deform the structure, even though this could be influenced by the chemical fixation. The confocal data provided qualitative evidence of the cell attachment and interaction with the microstructure environment. Cells were seeded at varying densities ranging from 5000 to 15,000 cells cm^−^^2^ to identify optimal culture conditions (Figure 5A–C). Analysis of the acquired confocal z‐stacks revealed that cells readily migrated toward the beam structures and “bridged” the inter‐beam regions. To ensure reliable mechanical readout, it is critical to maintain a low seeding density, thereby minimizing the likelihood of multiple cells simultaneously interacting with and deforming a single beam. Notably, cells exhibited the ability to mechanically engage with and deform the beams, indicating effective cell–scaffold coupling. Figure 5D,E presents a 3D reconstruction of a representative cell spanning between adjacent beams, highlighting its capacity to bridge structural elements within the scaffold.

**FIGURE 5:**
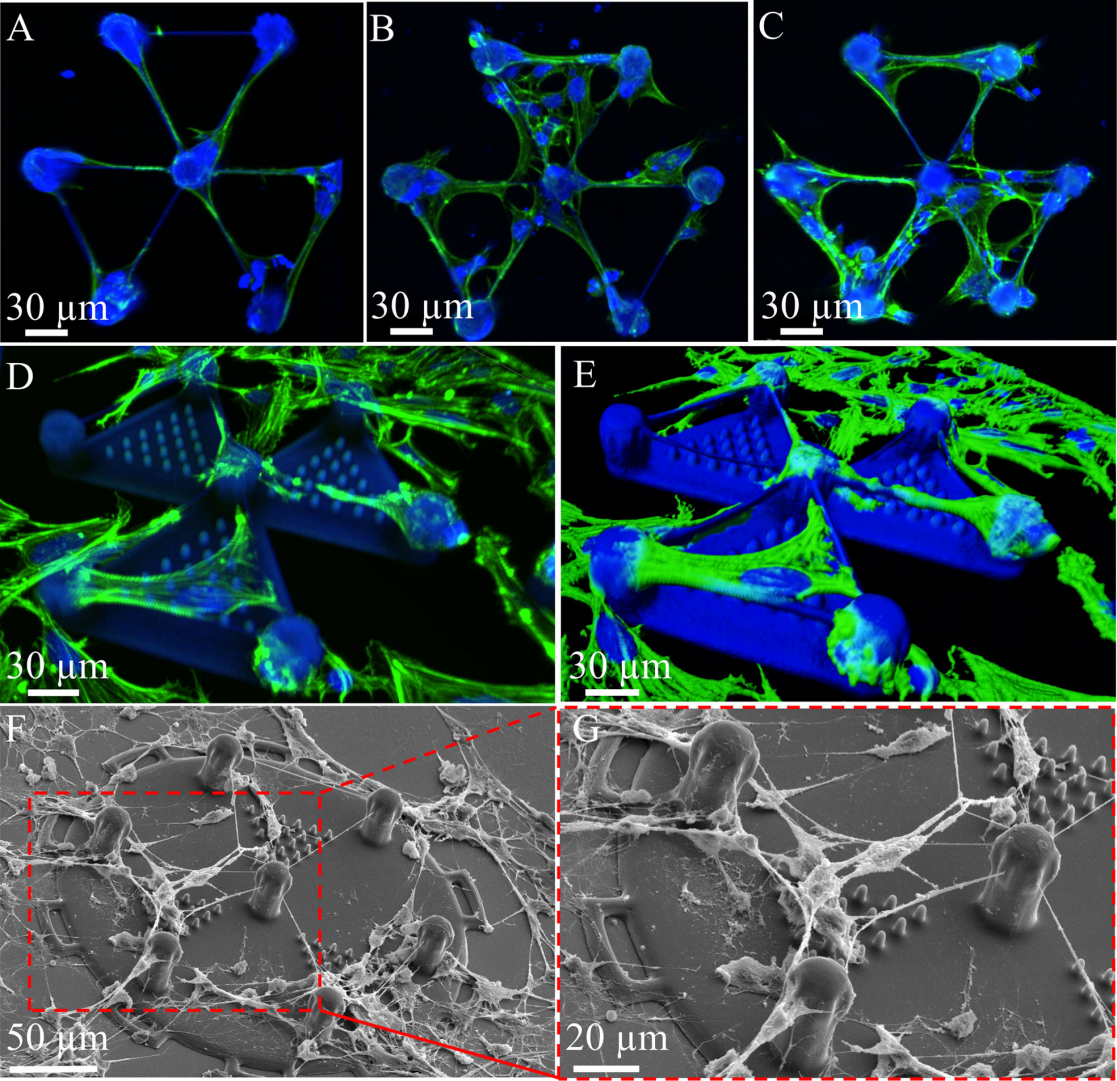
Confocal fluorescence and scanning electron microscopy (SEM) images of fixed NES cells cultured on the 3D scaffold. F‐actin was stained to visualize the cytoskeleton, and nuclei were counterstained with DAPI (A–E). (A–C) Confocal images at increasing seeding densities of 5000 (A), 10,000 (B), and 15,000 cells cm^−^^2^ (C), respectively. (D) Three‐dimensional confocal reconstructions of cell‐biomaterial interactions at low seeding density. (E) Rendered view of the 3D reconstruction shown in (D). (F, G) SEM images of dehydrated cells on the scaffold at low seeding density, highlighting cell morphology and cell–scaffold interactions.

**FIGURE 6:**
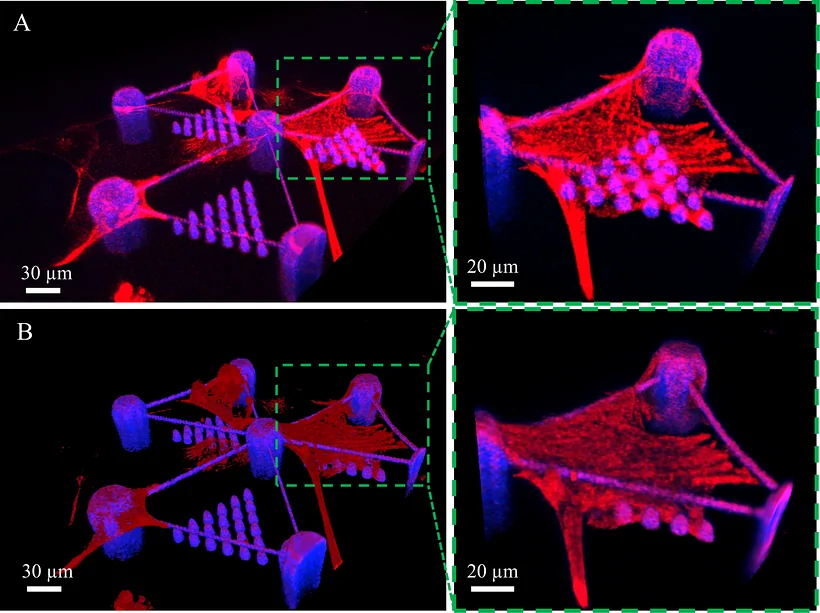
Three‐dimensional confocal z‐stack visualization of a representative time point (44 h) during live‐cell imaging. The red channel corresponds to F‐actin staining using SPY650‐FastAct, highlighting the cytoskeletal organization of the cells, while the blue channel represents the intrinsic autofluorescence of the printed IP‐PDMS scaffold, used for deflection measurements. (A) Maximum intensity projection (MIP, Imaris) of the z‐stack, providing an overview of cell morphology and spatial distribution on the scaffold. (B) Imaris volume rendering in blend mode, enabling visualization of the 3D arrangement of cells relative to the beam and micropillar structures. Insets show magnified views of the highlighted regions, illustrating cell spreading, attachment to the beams, and interaction with the micropillar array. Videos S3– S8 show the time‐lapse of this sample recorded for the live experiment.

After a secondary fixation using osmium tetroxide, the samples were subjected to a graded dehydration series using ethanol (30%–100%) to preserve cellular morphology as much as possible. Finally, a graded series of hexamethyldisilazane (HMDS) in ethanol was employed to prevent collapse of delicate cellular structures. The dried samples were then sputter‐coated with a thin conductive gold layer (∼5–10 nm) to minimize charging during imaging. SEM micrographs revealed detailed information on cell adhesion and 3D growth at high‐resolution. Cells were observed to attach to the scaffold surfaces, exhibiting characteristic spreading behavior and growing between the structural elements of the 3D print. In several instances, cells spanned across adjacent beams, indicating mechanical interaction (Figure 5F,G). It should be noted that the dehydration and drying steps inherent to SEM sample preparation may introduce artefacts, including cell shrinkage, cytoskeletal collapse, and slight alterations in beam morphology, which should be considered when interpreting morphological (cell) features in SEM images.

The combined confocal and SEM analyses demonstrated that the scaffold supports cell attachment and promotes spreading, with cells adapting their morphology to the underlying 3D IP‐PDMS architecture. Confocal imaging and SEM confirmed the physical interactions and anchoring of cells on the printed structures as well as the ability to induce beam deflection, even though the ones reported in SEM pictures can also be exacerbated by the necessary fixation and dehydration protocol. Together, these observations indicate that the 3D platform is suitable for further studies involving live‐cell imaging and mechanical analysis.

### 2.4 (Live) Confocal Microscopy and Cell Force Analysis

Live confocal microscopy was used to record the deflections of the beams in real time over 64 h during cell‐beam interaction and mechanical loading. To visualize cellular morphology under live conditions, a nontoxic, cell‐permeable, and highly specific F‐actin probe (SPY650‐FastAct, Spirochrome) was employed. This fluorogenic probe selectively labels filamentous actin, enabling imaging of the cytoskeleton without compromising cell viability. In combination with the intrinsic autofluorescence of the printed IP‐PDMS structures, this approach allowed simultaneous visualization of both cells and scaffold without the need for additional biomaterial labeling. Figure 6 presents a maximum intensity projection and a 3D volume rendering (blend mode) of cells on the scaffold at 44 h, revealing infiltration of the structure. Cells adhere to and span across the beams, micropillars, and pedestal, indicating active interaction with the 3D architecture. By acquiring images in separate fluorescence channels, the cytoskeleton of the cells and the geometry of the underlying microenvironment could be resolved, facilitating accurate assessment of cell–structure interactions over time. Beam deflection was quantified through post‐processing of the acquired image sequences using a custom semi‐automated MATLAB routine. This routine is based on an original idea of J. Kokorian et al. [54] and is explained in detail in the Supporting Information Figure S11. Time‐lapse videos of scaffolds with cells deforming the beams can be found in Videos S3–S8.

As illustrated in Figure 7, maximum intensity projections of the confocal z‐stacks were used to separately visualize the cellular actin network (red channel) and the scaffold geometry (blue channel). The analysis was performed exclusively on the structural (blue) channel to avoid interference from cellular fluorescence. A more detailed explanation of the force‐analysis pipeline is provided in Figures S11 and S12. First, two reference points were manually selected at the endpoints of the beam to define its initial axis. Subsequently, a series of equidistant cross‐sectional lines perpendicular to the initial beam axis were generated along its length (Figure 7E,F).

**FIGURE 7:**
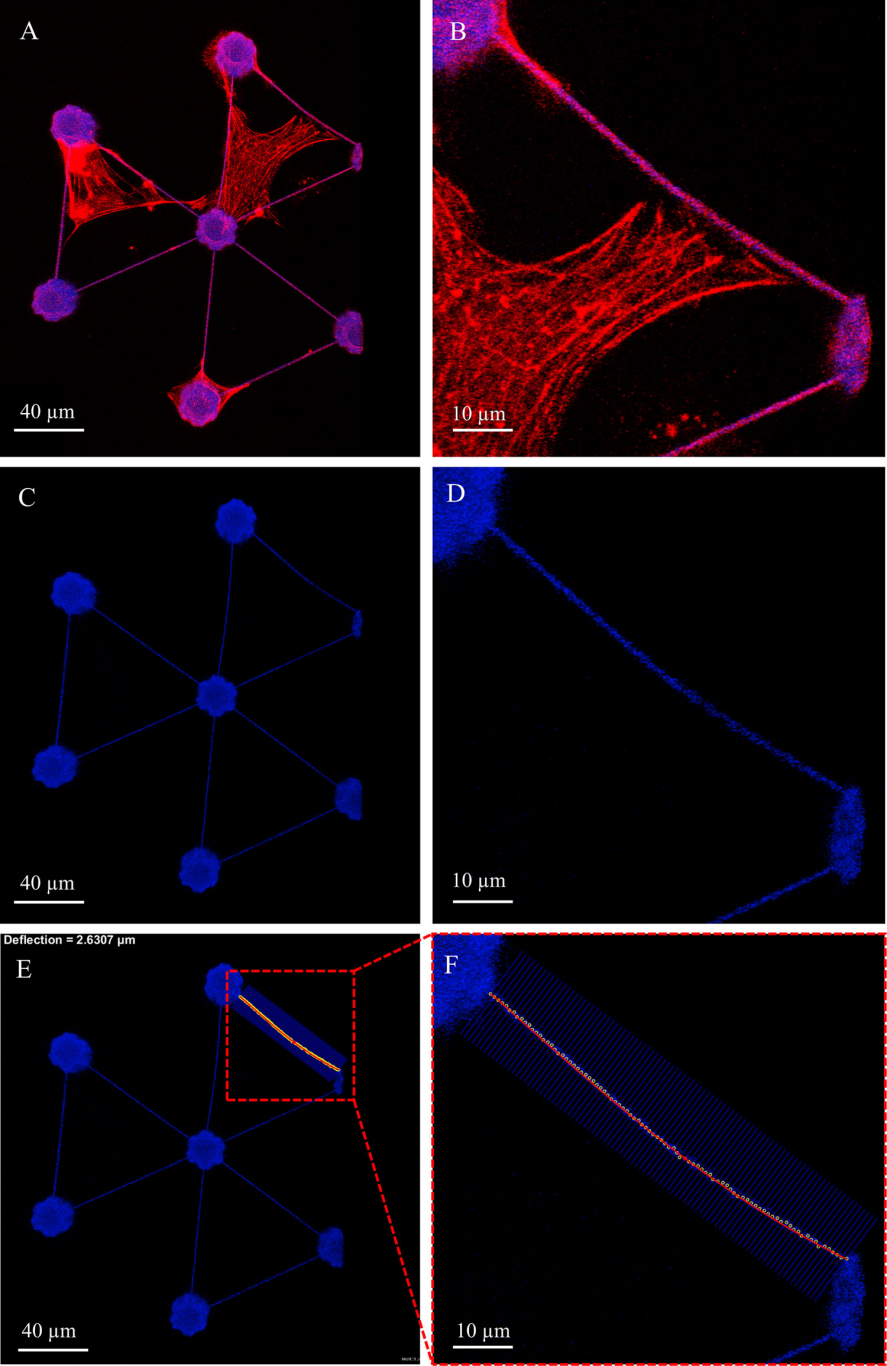
Workflow for beam deflection quantification from live confocal microscopy. (A,B) Merged fluorescence images showing F‐actin (red) and scaffold autofluorescence (blue), and illustrating a cell spanning between adjacent beams. (C,D) Isolated blue channel used for deflection analysis, enabling clear visualization of beam geometry without interference from cellular fluorescence. (E,F) Output of the semi‐automated MATLAB analysis. (E) Overview of the analyzed structure with the selected beam highlighted and fitted centerline (yellow) overlaid. (F) Magnified view showing the perpendicular intensity sampling lines (dark blue lines) and fitted deflection profile (red), used to determine the maximum beam deflection (δ ≈ 2.63 µm).

For each cross‐section, the fluorescence intensity profile along the sampling line was extracted, and the beam centerline was determined by identifying the dominant intensity peak corresponding to the scaffold signal. To improve spatial accuracy, a local parabolic fit was applied around the peak, enabling subpixel localization of the beam position. The resulting centroid positions were then transformed into a local coordinate system aligned with the beam, yielding the transverse displacement profile along its length. A cubic polynomial was fitted to these discrete points to obtain a continuous representation of the beam deflection (Figure 7E,F, yellow dots with red fitted curve; more details in Figure S11).

The maximum deflection was extracted from the fitted curve after correcting for boundary offsets, and converted from pixel units to micrometers by extracting the size of the scale bar. This workflow enables robust and reproducible quantification of beam bending from confocal datasets, allowing direct correlation between observed cell and the resulting mechanical deformation of the beam.

The magnitude of these deflections and their respective position along the beam were used to calculate the forces exerted by single cells based on the known spring constant. The spatial resolution of the deflection measurements allowed precise mapping of force application points and bending. Remarkably, the measurements demonstrate that our setup enables reliable detection of forces in the nN range, confirming the sensitivity and robustness of the method for quantifying cellular mechanical behavior. It is noteworthy to mention that, as cells migrate away from the beams, the beams exhibit a clear elastic recoil toward their undeformed position, demonstrating that the platform can resolve dynamic changes in cellular loading and unloading over time. This behavior is illustrated in Figure S13, where beam recovery is observed over a 2 h interval between consecutive images. This behavior is consistent with the expected mechanical response of the cantilevers upon release of cell‐generated traction forces.

All measurements were performed across multiple independent samples (control: *n* = 5; TSC2: *n* = 3), with a total of 100 and 65 individual deflection events analyzed for each group, respectively. The obtained data were subsequently blind analyzed to characterize cellular forces of the control (i.e., healthy) and TSC2. Even though loss of TSC2 function has been shown to cause cytoskeletal dysregulation in neural cells [23, 24, 26], no significant changes in force generation were observed between healthy and TSC2 cells under the tested conditions (Figure 8), and force distributions displayed substantial cell‐to‐cell variability within each group, consistent with previously reported heterogeneity in NES cells mechanical behavior and force development over longer culture times [55]. This behavior evolves over culture time and depends on differentiation state and microenvironmental cues. For example, AFM‐based studies have shown that NPCs, neurons, and glial cells display distinct elastic moduli and membrane mechanics within the same population, reflecting functional and phenotypic diversity [55]. Specifically, healthy control cells exhibited a mean beam deflection (in plane) of 2.46 ± 1.27 µm (median = 2.25 µm, *n* = 100), corresponding to an applied force of 6.50 ± 3.41 nN (median = 5.58 nN, *n* = 100), whereas TSC2 cells showed a comparable deflection of 2.35 ± 1.33 µm (median = 1.82 µm, *n* = 65), corresponding to a force of 6.35 ± 3.34 nN (median = 5.15 nN, *n* = 65). This suggests that either the mechanical signatures (i.e., exerted forces) of these cells remain preserved despite cytoskeletal alterations, or that compensatory mechanisms may stabilize force production. The relatively large standard deviations observed in the data likely arise from donor‐to‐donor variability between individual control and patient‐derived cell lines. Each individual line represents indeed a distinct genetic and phenotypic background, which can lead to differences in cellular behavior and mechanical properties. In addition, variability may also occur between otherwise identical cells derived from the same donor, as individual cells may exert forces of differing magnitudes and may not all operate at their maximum mechanical capacity. An alternative explanation is that cells might regulate displacement rather than force; in this case, cells would generate approximately similar forces provided these remain within their mechanical limits. To test this hypothesis, further experiments using beams with varying stiffness would be required. When measurements from multiple donors are pooled within a group, this inter‐donor heterogeneity contributes to increased spread in the data, as shown in Figure S14 reporting the beam deformation and force boxplots grouped per control and TSC2 cell line. In addition to hypothesis testing, effect sizes (Cohen's term *d*) and 95% confidence intervals were calculated to quantify the magnitude and precision of the observed differences. Both comparisons yielded negligible effect sizes (maximum bending: *Cohen's d* = −0.09; traction force: *Cohen's d* = −0.04), small confidence intervals (maximum bending, 95% CI: −0.53 to 0.29 µm; traction force, 95% CI: −1.20 to 0.91 nN), indicating that the observed differences were small relative to the variability within each group. These results further support the conclusion that CNTRL and TSC2 cells exhibit similar mechanical behavior under the conditions investigated.

**FIGURE 8:**
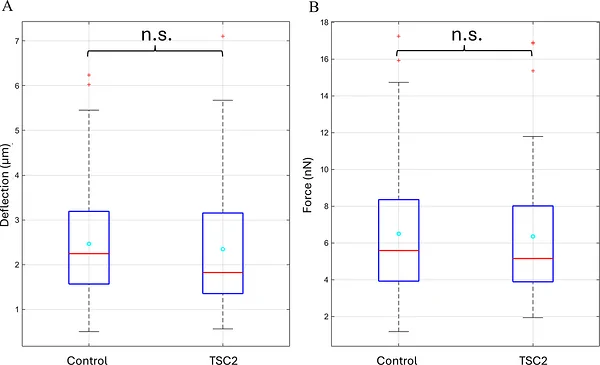
Quantitative comparison of cell‐induced beam deformation and corresponding forces (in plane) for control and TSC2 cells. Boxplots represent the interquartile range (IQR) with the median indicated by the red line, whiskers showing the full data range excluding outliers, and crosses denoting outliers. Mean values are indicated by cyan colored circles. (A) Distribution of measured beam deflections. For the control group, the median deflection (IQR) was 2.25 µm, and the mean was 2.56 ± 1.27 µm (*n* = 100), while for the TSC2 group the median deflection was 1.82 µm and the mean was 2.34 ± 1.33 µm (*n* = 65) with *p* = 0.65. (B) Corresponding calculated forces derived from beam bending analysis. For the control group, the median force was 5.58 nN, and the mean was 6.50 ± 3.41 nN (*n* = 100), while for the TSC2 group the median force was 5.15 nN and the mean was 6.35 ± 3.34 nN (*n* = 65). The force plot takes into account the location of the equivalent force on the beam. Both groups exhibit comparable distributions in deflection and force, highlighting similar mechanical interaction with the beam. Maximum bending: Cohen's *d* = −0.09, 95% CI: −0.53 to 0.29 µm; traction force: Cohen's *d* = −0.04, 95% CI: −1.20 to 0.91 nN.

Specifically, although TSC2 mutations are known to disrupt cytoskeletal organization, the preserved force output observed in our study suggests that compensatory mechanical regulatory mechanisms may be at play. Cellular contractile output depends primarily on actomyosin contractility, which is principally governed by RhoA‐mediated activation of ROCK, leading to phosphorylation of myosin light chain (MLC) to drive actomyosin‐based force generation [56]. In line with this, Catlett et al. demonstrated that TSC2 haploinsufficiency impairs RhoA activation in iPSC‐derived neurons through a direct, mTOR‐independent mechanism [26], suggesting that partial TSC2 loss could reduce contractile output. However, this effect may be counteracted by the consequences of mTORC1 hyperactivation, since mTORC1 can regulate RhoA at the level of protein translation [57], thereby increasing the total pool of RhoA available for activation. Furthermore, mTORC1 activity has been shown to promote focal adhesion maturation through FAK phosphorylation [58], potentially enhancing the efficiency of force transmission independently of actin network organization. Together, these opposing and interconnected influences create a complex regulatory landscape. Moreover, these effects have largely been described in differentiated neuronal cells, and regulation in NES cells may differ. Further studies directly measuring RhoA‐GTP levels and MLC2 phosphorylation in TSC2 ± NES cells will be necessary to better understand the regulation of contractile force generation.

In this study, we included five control cell lines and three patient‐derived cell lines. Due to inter‐donor variability, substantial heterogeneity was observed within each group, resulting in relatively large standard deviations when all measurements were pooled. This variability reflects biological differences between individual cell lines and experimental noise. To account for this, analyses were complemented with per‐patient approaches and mixed‐effects models that explicitly consider patient‐specific variation. Furthermore, the observed substantial cell‐to‐cell variability in terms of force distributions could also reflect differences in cell cycle stage, adhesion, maturation, cytoskeletal organization, and the cell‐beam attachment area. These results underscore that cytoskeletal morphology and net traction force magnitude are related but not directly proportional parameters, and should be interpreted as distinct aspects of cellular mechanobiology.

In addition to in‐plane force measurements derived from lateral beam deflection, the platform enables quantification of out‐of‐plane (*z*‐direction) deformations using the confocal z‐stacks. Due to the limited number of samples exhibiting bending in the *Z‐*direction, a statistically robust analysis could not be performed and was therefore omitted from this study. Furthermore, instances of *Z*‐direction bending often involved contributions from multiple cells, complicating the interpretation. Nevertheless, as a proof of concept, we demonstrate that visualization of bending in the *Z*‐direction is feasible. By acquiring volumetric image stacks of the scaffold–cell system, the 3D geometry of the beams can be reconstructed, allowing direct visualization of vertical displacement induced by cellular forces. As shown in Figure 9, comparison of z‐stack projections at different time points reveals measurable deformation of the suspended beam along the *z*‐axis. In the undeformed or minimally loaded state, the beam exhibits a relatively straight profile between the supporting pillars (Figure 9A,B). In contrast, under cellular interaction, a clear downward displacement of the beam is observed, resulting in a curved profile indicative of out‐of‐plane bending (Figure 9C,D). The use of confocal microscopy provides sufficient axial resolution to resolve micron‐scale height differences, enabling extraction of beam profiles along the *z*‐direction. Similar to in‐plane analysis, this approach allows determination of maximum deflection and its spatial location.

**FIGURE 9:**
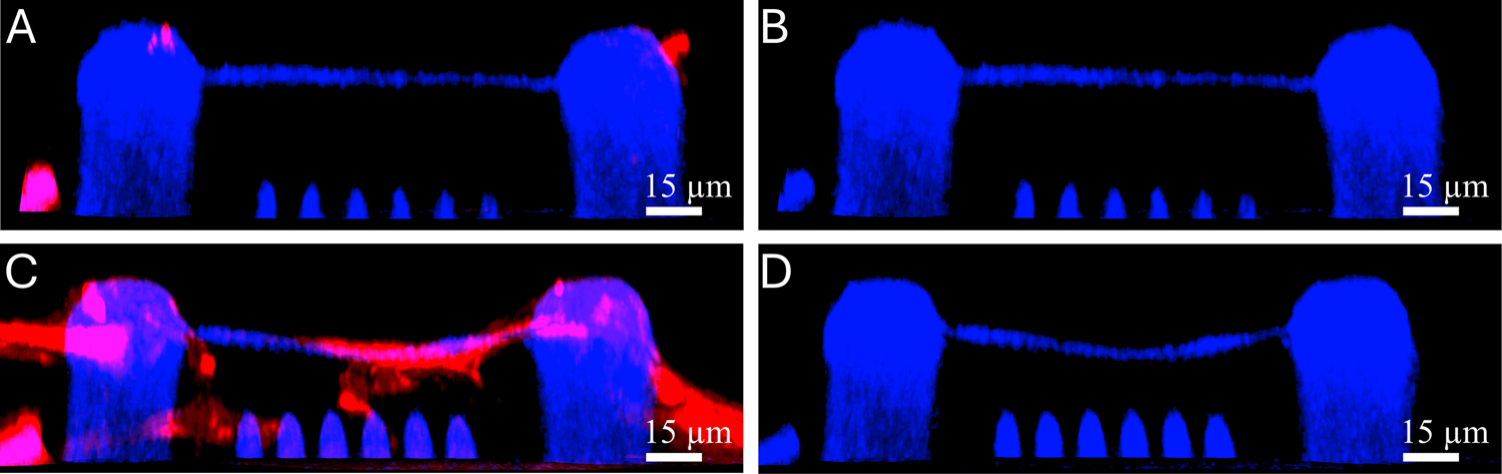
Confocal z‐stack projections (side view) illustrating out‐of‐plane deformation of suspended beams induced by cell activity over time (bending in *z‐*direction). (A,B) Representative images after 24 h of imaging, showing minimal beam deflection between the supporting pillars. (C,D) Corresponding images after 48 h, revealing pronounced downward bending of the beam, indicative of increased cell‐induced mechanical loading. Red channel (A,C) highlights the live actin marker and cellular morphology, while the blue channel (B,D) provides complementary visualization of the 3D‐printed structure.

Importantly, the ability to measure both in‐plane and out‐of‐plane deflections provides a more comprehensive characterization of cell‐generated forces. While lateral deflection primarily reflects traction forces parallel to the substrate, vertical bending captures components of force associated with cell contraction, adhesion, and 3D interaction with the scaffold. This showcases the capability of the platform to probe complex cellular force generation in a fully 3D environment. Due to the higher cross‐sectional moment of inertia in the *z*‐direction, the bending stiffness of the beam is increased in this orientation, requiring larger forces to induce deformation. Consequently, out‐of‐plane bending by a single cell was only rarely observed. In contrast, more pronounced bending in the *z*‐direction occurred when multiple cells were attached to the same beam.

Compared to hydrogel‐based 3D TFM [29], the presented 2PP elastomeric platform offers several practical and analytical advantages. First, the mechanical properties of the sensing elements are defined by geometry and directly measurable via AFM, eliminating uncertainties related to hydrogel swelling, degradation, or batch variability. Second, force readout relies on deterministic beam mechanics rather than inverse computational reconstruction of distributed matrix deformations, significantly simplifying data analysis. Third, the printed microarchitecture can simultaneously guide cell attachment and enable force measurements in three dimensions. Together, these features ensure reproducibility and make the system particularly suitable for comparative mechanophenotyping.

Some limitations should also be acknowledged. The current design primarily enables measurement of in‐plane beam bending. While out‐of‐plane forces can also be assessed, the higher bending stiffness in this direction reduces sensitivity, and such deformations are therefore less frequently observed. Additionally, force transmission depends on stable focal adhesion formation at the beam surface, which may vary between cells. The measurement represents net force applied at a specific beam location and does not capture subcellular force heterogeneity. Furthermore, while live imaging was performed over extended periods, long‐term culture beyond 60 h was not evaluated. Addressing these aspects in future iterations may further improve physiological relevance and multidirectional force detection.

The measured average forces (∼6 nN) are consistent with previously reported traction forces of neuronal and progenitor cell types measured in 2D systems (5–11 nN) [30]. It is important to note that the measured force exerted by the cells on the beams was approximated as an equivalent point load. In reality, however, the applied force is more accurately described as a distributed load along the contact region. Because cells typically adhere to the suspended beams over an area rather than a single point, we further evaluated the validity of the equivalent point‐load assumption using FEM. These simulations compared a concentrated point load to a uniformly distributed load applied over a 20 µm area. Simulations were performed for both an idealized uniform beam and a nonuniform beam geometry incorporating fabrication‐induced defects (i.e., 120 nm imperfections) on the top and bottom of the beam, see Figure S15. The distributed load resulted in a maximum beam deflection of only 4%–5% lower than that predicted by the equivalent point‐load model, while the influence of beam nonuniformity was less than 1%. These results indicate that the equivalent point‐load assumption provides a reasonable first‐order approximation for estimating cellular traction forces in the present system. A detailed description of the FEM analysis, including the loading conditions, beam geometries, deformation profiles, and quantitative comparison, is provided in Figures S15, S16 and Table S1. Overall, our findings suggest that NES cells maintain comparable global contractile output in 3D microstructure environments, despite differences in geometry and adhesion configuration. Importantly, the magnitude observed here, reflects collective actomyosin contractility transmitted through adhesion points rather than distributed substrate traction, reinforcing the concept that NES cells operate within a tightly regulated mechanical force window during development.

## 3 Conclusions

In this work, we introduce an elastomeric, mechanically characterized 3D force‐sensing platform fabricated through 2PP of IP‑PDMS combined with a controlled wet‑etching protocol. This two‐step approach enabled the fabrication of submicron, compliant beams with excellent bending sensitivity while maintaining structural integrity and chemical stability. By combining these engineered microstructures with live confocal imaging and sub‑pixel MATLAB‐based deflection analysis, we achieved reliable quantification of cell‑generated forces in the single‑nN range within a fully 3D environment.

We demonstrate that human iPSC‑derived NES cells readily adhere to and deform the printed structures, enabling continuous force measurements over extended imaging periods. Despite known cytoskeletal abnormalities associated with TSC2 mutations, force output from TSC2‑derived NES cells did not significantly differ from healthy controls under the tested conditions. This finding suggests that either baseline mechanobiological force generation is preserved in these cells or that compensatory mechanisms stabilize force production despite cytoskeletal alterations. Improved etching control through automation, optimization of etchant concentration, or possibly through CF_4_ or SF_6_ dry etching, could improve the yield even more. In addition, the drying process potentially influences yield as capillary forces in wet‐etching processes can pull the beams toward the substrate, limiting the achievable beam stiffness. High humidity environments cause condensation of water on the NOVEC 7100 [59], and TBAF is extremely hygroscopic, altering the etching properties. Leaving the substrates in deionized water after etching could prevent collapse as well.

Overall, the platform provides a sensitive, reproducible, and physiologically relevant method for probing neural cell mechanobiology in 3D. Beyond enabling direct comparisons between healthy and disease‑model neural cells, the approach offers broad potential for studying intracellular force dynamics, ECM–cell mechanical interactions, and mechanotransduction pathways. With its high sensitivity and compatibility with live imaging, this system is a meaningful step toward investigating biophysical mechanisms in models of neurological disorders. In this regard, TSC was selected as a relevant proof‐of‐concept model given its well‐described cytoskeletal defects and its nature as a prototypic mTOR pathway disorder. Importantly, mTOR dysregulation extends beyond TSC and represents a convergent pathogenic mechanism in a broad range of neurological disorders, including Alzheimer's disease, Parkinson's disease, and amyotrophic lateral sclerosis [60, 61]. The absence of significant differences between TSC2‐mutant and control cells likely reflects donor variability and the possibility that cells regulate displacement rather than force. Future experiments could decouple force and displacement regulation, as well as include pharmacological inhibition of cytoskeletal contractility to directly validate the platform's sensitivity to force perturbations. Future studies could also further extend the platform to glial cell types [62], increasingly recognized as active contributors to neurological disease. Additionally, larger cohorts of patient lines and additional disease types could help reveal more subtle mechanophenotypic differences. The ability to reliably measure nN‐scale forces in live NES cells within a defined 3D microenvironment provides a quantitative mechanical phenotype that can complement molecular readouts. This platform may be particularly valuable for screening pharmacological modulators of mTOR signaling, cytoskeletal regulators, or mechanotransduction pathways. Changes in force magnitude, dynamics, or attachment stability could serve as early functional biomarkers preceding morphological or other functional alterations, thereby expanding the toolkit for mechanobiology‐informed therapeutic development.

## 4 Experimental Section

### 4.1 Two‐Photon Polymerization Setup Configuration

The design of the 3D microenvironment comprises three distinct parts: a pedestal with micropillars, large support columns, and thin force‐sensitive beams. Both the pedestal and columns were designed in CAD software SolidWorks 2023 (Dassault Systems SolidWorks Corp., Vélizy‐Villacoublay, France). The slicing was performed similar to the workflow, as described in our previous work [37]. Summarized, the CAD files were sliced in DeScribe (Nanoscribe GmbH & Co. KG, Karlsruhe, Germany), after which the printing parameters were set in separate job files. The slicing and printing parameters were optimized using dose tests for every part of the design and are listed in the Supporting Information (Table S2). The main print job combined the individual print jobs of a pedestal, six support columns, and nine force‐sensitive beams (Figure 1). The force‐sensitive beams were printed suspended 37 µm above the substrate with a length of 75 µm and were programmed as voxel trajectories instead of using a CAD file. These trajectories are perpendicular to the longitudinal axis of the beam with a length of 0.8 µm.

For the fabrication of the 2PP structures, a commercial two‐photon direct laser writing setup was employed (Photonic Professional GT+ system, Nanoscribe GmbH & Co. KG, Karlsruhe, Germany) with a femtosecond fiber laser source with a wavelength of 780 nm, a standard maximum laser power of 50 mW (corresponding to 100% laser power), a repetition rate of 80 MHz, and a pulse length of 100 fs. A LCI Plan‐Neofluar 25×/0.8 Imm Corr DIC M27 (Carl Zeiss, Oberkochen, Germany) microscope objective was employed in dip‐in laser lithography (DiLL) mode during the fabrication (i.e., with the lens dipped directly in the photopolymer).

All structures were printed on 25 × 25 × 0.7 mm3 (*l* × *w* × *h*) indium tin oxide (ITO)‐coated soda–lime substrates. To maximize the output, each substrate was divided into quadrants by inscribing them before printing on the ITO side, using a precision top scribing tool (PELCO FlexScribe 200, Ted Pella Inc., Redding, California), allowing the printing of four sets of prints per substrate. Before printing, the substrates were first cleaned with acetone using a lint‐free wipe. The samples were then cleaned and the surface activated using an oxygen plasma setup (Diener electronic GmbH + Co. KG) for 30 min at a power of 80 W and an oxygen gas flow rate of 5 cm^3^/min. The adhesion of the prints to the substrate was improved by silanizing the substrate with 3‐(trimethoxysilyl)propyl methacrylate (MAPTMS). A drop of MAPTMS was deposited on parafilm, after which the plasma‐cleaned substrates were put on top with the ITO surface facing toward the MAPTMS. The substrates were then left for at least 16 h before using them for printing. Before printing, the substrates were cleaned with acetone and a lint‐free cloth to remove excess MAPTMS.

A drop of IP‐PDMS was cast on top of each quadrant of the substrate and one in the center. In addition, a drop of IP‐PDMS was deposited on the objective in the machine to ensure proper approach and automatic interface finding. Nine substrates could be printed in series, yielding a total of 36 samples per print. Each of these samples had nine separate structures printed. Different writing parameters were employed for each part of the print, optimized to meet the different requirements. The writing parameters can be found in Table S2.

After the 2PP process was finished, all samples were chemically developed in isopropanol for 15 min to remove the unexposed IP‐PDMS, followed by a second rinse of 1 min in clean isopropanol. The samples were then immersed in NOVEC 7100 engineered fluid to prevent their collapse due to surface tension when drying. All samples were left to air dry under a fume hood. It is important to control the humidity of the environment as water can condense on the NOVEC, after which the structures can collapse (observed for prints developed in high relative humidity >65%) [59]. While the beams are designed to be straight, they appear curved during printing and development in IPA, after which they straighten in NOVEC and remain straight following drying (Figure S1).

### 4.2 TBAF Wet Etching

To reduce the feature sizes of the beams, TBAF wet etching was employed. To prevent variability due to humidity, a new batch was prepared for each etching session. The whole protocol was carried out under a chemical fume hood. First, DMSO was pipetted into a dark vial, after which the TBAF hydrate (TCI chemicals, 22206‐57‐1) was weighed and added to the vial to achieve concentrations of 0.88 M (2.79 g in 10 mL) and 0.57 M (1.81 g in 10 mL) for testing etching performance and for cell culture experiments, respectively.

The samples were vertically immersed in the etchant to allow any residue to be removed from the structure. The etch time was controlled by a stopwatch. After the intended etch time, 60 s for the cell experiments and variable for the etch performance test, the samples were immediately immersed in fresh isopropyl alcohol (IPA) to terminate the etching process in a horizontal position. After 2 min, the samples were transferred to a fresh batch of IPA for another 2 min, followed by two subsequent rinses in NOVEC 7100 for 1 min each. The samples were then left vertically to dry in air.

After etching, samples were inspected using an optical microscope. Subsequently, a Grace Bio‐Labs SecureSeal imaging spacer (9 mm diameter well, 120 µm thickness) was mounted onto the sample. The spacer enabled inversion of the sample during live imaging in a well plate, compatible with the use of an inverted confocal microscope.

### 4.3 Scanning Electron Microscopy

Samples were first coated with a thin conductive gold layer prior to imaging to prevent charging effects. Sputter coating was performed using a JEOL JFC‐1300 Auto Fine (JEOL Ltd., Tokyo, Japan), a low‐voltage planar magnetron sputter coater operated with argon as the sputtering gas. The samples were sputtered twice at 30 mA for 30 s at a working distance of 25 mm from the target—once normal to the surface and once at a 45° tilt—to ensure uniform coverage. This procedure resulted in an approximate gold layer thickness of ∼10–30 nm.

SEM imaging was carried out using a JEOL JSM‐6010LA (JEOL Ltd., Tokyo, Japan) under high‐vacuum conditions. Images were acquired in secondary electron imaging (SEI) mode at accelerating voltages ranging from 5 to 20 kV. Micrographs were acquired at both 0° and 90° tilt angles to examine cross‐sectional features.

To minimize charging effects during imaging, the top surfaces of the ITO‐coated substrates were electrically grounded to the SEM sample holder using conductive carbon tape, ensuring good electrical contact throughout the measurements.

### 4.4 Raman Spectroscopy

Raman spectra were acquired using a Horiba LabRAM S3000 Raman spectrometer equipped with a Horiba Synapse CCD detector. The measurements were performed using a 632.8 nm He–Ne laser as the excitation source and a 50× microscope objective. The spectrometer was configured with an 1800 lines mm^−^^1^ diffraction grating. Spectra were recorded in the 399–3500 cm^−^^1^ spectral range with an acquisition time of 5 s and 1 accumulation per spectrum. The detector was maintained at −132.8°C. The entrance slit and confocal hole were both set to 1000 µm, and no neutral density filter attenuation was used (ND filter 100%). The red excitation channel was selected for analysis, as the intrinsic autofluorescence of the material was limited and enabled reliable signal detection, while the green channel exhibited excessive autofluorescence that hindered accurate quantification.

### 4.5 Mechanical Characterization

#### 4.5.1 Beam Characterization

Mechanical characterization of the printed IP‐PDMS microstructures was performed using a JPK NanoWizard 4 AFM (Bruker/JPK Instruments, Berlin, Germany) mounted on an inverted optical microscope to enable precise positioning of the probe on individual beams. All measurements were conducted at room temperature in ambient air, in aqueous conditions, and in physiological imaging medium at 37°C. The compliance of the beam measured in an aqueous environment was used to calculate the forces exerted in the cell experiments.

Soft, tip‐less silicon nitride cantilevers (NuNano Quest T 0.005, NuNano Ltd., Bristol, UK) with a nominal spring constant of 0.005 N m^−^^1^ (manufacturer range 0.003–0.007 N m^−^^1^) were used to ensure high sensitivity when probing compliant microstructures. The exact spring constant of each cantilever was calibrated prior to measurement using the thermal noise method and the contact‐based method. The optical lever sensitivity was determined from force–distance curves acquired on a rigid ITO‐coated glass substrate to prevent electrostatic effects. To determine the effective spring constant of the suspended beams, force–displacement curves were acquired at the center of each beam using contact mode. The structures were fabricated at the edge of the substrate to enable force measurements in the *XY* plane, aligning with the main direction of cellular traction and in the direction of the highest sensitivity of the beam. For AFM measurements, the substrates were mounted at a 90° angle, and the indentations were performed on the side of the beam. The approach velocity was kept low to ensure quasi‐static loading conditions (0.1 ms^−^^1^) with a setpoint force of 1.2–2 nN and a Z length of 3–8 µm. For each beam, two measurements were carried out. The first measurement was performed on the ITO substrate to calibrate the cantilever response, followed by a measurement on the suspended beam to separate the compliance contributions of the cantilever and the structure, thereby minimizing systematic errors. The system was modeled as two linear springs in series. The force curves were recorded for multiple beams (*n* = 4). Data processing and stiffness extraction were performed using custom scripts in MATLAB (MathWorks, Natick, MA, USA).

Quantitative nanoindentation measurements were performed to determine the YM of the printed IP‐PDMS material. Two approaches were employed: AFM and interferometer‐based indentation.

#### 4.5.2 AFM

To determine the material properties of the printed structures, force mapping measurements were performed on beams that had collapsed onto the underlying ITO substrate. The collapse of the beam onto the substrate eliminates the need to consider the spring constant, thereby enabling direct determination of the material's YM. These measurements were conducted under ambient conditions to obtain representative mechanical properties of the printed IP‐PDMS material independent of beam bending effects. The collapsed beams provide a flat contact geometry that allows reliable indentation‐based characterization of the material. Force mapping was performed using qp‐BioAC cantilevers (Bruker AFM Probes) with a nominal spring constant *k* = 0.06 N m^−^^1^, a cantilever length of *L*  =  80 µ*m*, and a nominal tip radius of 10 nm. Prior to the measurements, the cantilever spring constant was calibrated. Measurements were carried out in QI mode over the beam surface, yielding a total of 713 individual force–indentation curves.

The obtained force–indentation curves were analyzed using a Hertz contact model implemented in the JPK data processing software. A Poisson's ratio of 0.5 was assumed for the elastomeric IP‐PDMS material. Fits were applied to the approach segment of each curve within the elastic deformation regime. The YM values obtained from the individual fits were subsequently averaged over all 713 measurement points to obtain the representative YM of the printed beams.

#### 4.5.3 Interferometer‐Based Nanoindentation

The interferometer‐based indentation measurements were performed on a solid pedestal (60 × 30 × 5 µm) printed with identical parameters as the beams. A Piuma nanoindenter (Optics11 Life b.v., Amsterdam, The Netherlands) was employed to measure the mechanical properties of the printed pedestal. A cantilever with a stiffness of 51.2 Nm^−^^1^ and a tip radius of 20 µm was used to perform a matrix scan yielding 25 measurements in air. Indentation depths were limited to 800 nm to prevent effects from the underlying substrate. The force–indentation curves were analyzed using a Hertzian contact model in the Piuma data viewer software. The indentation protocol is similar to our previous work [37].

### 4.6 Neural Progenitor Cell Culture

#### 4.6.1 Neuroepithelial Stem Cell Differentiation

The pre‐existing iPSC lines used in this study (see Table 1) were generated prior to this work and maintained according to previously described protocols [63]. iPSCs were differentiated into NES cells in N2/B27 medium supplemented with dorsomorphin and SB431. After 1–2 weeks in culture, neural rosettes appeared and were isolated by manual dissection. NES cells were then maintained in N2/B27 medium supplemented with fibroblast growth factor 2 (FGF2) and epidermal growth factor (EGF) [63].

**TABLE 1:** Preexisting cell lines employed in this study with line‐specific information.

Line #	Genotype	Gender	Age	Source
445	Control	F	11 y	Coriell Institute, Camden, NJ, USA; GM02036
446	Control	M	5 y	Coriell Institute, Camden, NJ, USA; GM05381
451	Control	M	19 y	Coriell Institute, Camden, NJ, USA; GM23973
420	Control	M	21 y	Coriell Institute, Camden, NJ, USA; GM23964
442	Control	M	50 y	Coriell Institute, Camden, NJ, USA; GM23970
417	TSC2	F	16 y	Coriell Institute, Camden, NJ, USA; GM03958
424	TSC2	F	26 y	Coriell Institute, Camden, NJ, USA; GM06102
447	TSC2	F	22 y	Coriell Institute, Camden, NJ, USA; GM06100

#### 4.6.2 Seeding Density and Culture Length Optimization

To determine the optimal cell density and culture duration for obtaining a reliable mechanical readout, NES cells were plated at varying densities of 5000, 10,000, and 15,000 cells/cm^2^, and cultured for 48, 72, or 96 h in N2/B27 medium supplemented with FGF2 and EGF. The medium was replaced with growth factor‐free medium 24 h after passaging, and cells were fed every other day thereafter.

#### 4.6.3 Culture of NES on 3D Microenvironments

To study cell‐generated forces, NES cells were plated at a density of 10,000 cells/cm^2^ in N2/B27 medium supplemented with FGF2 and EGF. After 24 h, F‐actin filaments were stained with the live‐cell imaging probe SPY650‐FastAct (Spirochrome) in growth factor‐free medium and maintained in the same medium during confocal live‐cell imaging for the entire duration of the experiment.

### 4.7 Confocal Imaging

#### 4.7.1 Fixed Cell Confocal Imaging (Cell Culture Conditions Optimization)

Samples were fixed in 2% paraformaldehyde (PFA) to preserve cellular morphology and cytoskeletal organization. Following fixation, cells were stained with ReadyProbes Reagent F‐Actin Phalloidin Conjugates 488 (Catalog number R37110, Thermo Fisher Scientific) to label filamentous actin and DAPI to visualize cell nuclei.

Confocal imaging was performed using an upright spinning disk confocal microscope (Andor Dragonfly 200, Oxford Instruments). The system was equipped with a 25× water‐dipping apochromatic objective (Nikon CFI75 Apo 25×, NA 1.19, working distance 2 mm), enabling high‐resolution imaging of the 3D scaffold environment.

Fluorescence excitation and detection were carried out using appropriate laser lines and emission filters. Phalloidin‐stained F‐actin was imaged using a 488 nm excitation laser with emission collected at 521 nm (GFP channel), while DAPI‐stained nuclei were excited at 405 nm with emission collected at 445 nm. The 405 and 488 nm laser lines were operated at 1.0% and 100 ms exposure time and 1.1% intensity and 120 ms exposure time, respectively. Images were acquired under identical acquisition settings for all samples to ensure comparability.

Z‐stacks were recorded with 200 nm steps in z to capture the full 3D architecture of the cell–scaffold system. The stacks were loaded in Imaris for analysis through maximum intensity projections, and 3D renderings were generated for visualization.

#### 4.7.2 Live‐Cell Confocal Imaging (Study of Cell‐Generated Forces)

Samples were mounted on the microscope stage under physiological conditions, at 37°C and 5% CO2 in humidified conditions to ensure cell viability throughout imaging. The autofluorescence of the 2PP printed structures in the blue and green channels enabled high‐contrast visualization of beam deformation and cell attachment. The cells were stained with a live actin stain that emits in far red (SPY650‐FastAct, Spirochrome). Time‐lapse z‐stacks were acquired using an inverted laser‐scanning confocal microscope (Nikon Ti2 AXR enclosed within an Oko Labs environmental chamber) equipped with a high numerical aperture objective to achieve sufficient spatial and temporal resolution. Z‐stacks were collected at defined intervals to capture 3D deformation of the beams over time. Imaging parameters, including laser intensity, scanning speed, and detector gain, were optimized to minimize photobleaching and phototoxicity while maintaining adequate signal‐to‐noise ratio (listed in Table S3).

These z‐stacks were acquired over a period of 64 h in nine different wells with three stacks per well, consisting of five control and three TSC2 cell lines. During the first 16 h, wells were imaged every 4 h; during the next 28 h, they were imaged every 2 h; and during the final 20 h, they were imaged every 4 h again. The time‐lapse stacks were converted to Imaris format for analysis due to the large file size. Each time‐lapse was assessed by a blinded assessor, and z projections of the region of the bending beams with only one cell deforming it were saved as PNG files. Quantification of forces was conducted exclusively for time points exceeding 20 h. Measurements prior to this time point were not included in the analysis to ensure that the cells had reached a stable state before mechanical characterization.

### 4.8 SEM Imaging of Dehydrated Samples

For SEM, the fixed cell‐seeded scaffolds were subjected to a dehydration protocol to preserve cellular morphology and structural integrity. All preparation steps were performed in a fume hood unless stated otherwise. Samples were first washed three times to remove residual fixative. A secondary fixation step was then carried out using 1% osmium tetroxide (TCI Chemicals, O0650) in deionized water for 1 h at room temperature to enhance the contrast of the membrane in the SEM and stabilize cellular structures. Following fixation, samples were rinsed once with PBS.

Dehydration was performed through a graded ethanol series by sequential immersion in 30%, 50%, 70%, 90%, and 100% ethanol (in demineralized water) for 4 min each. As the next step, to minimize structural collapse due to surface tension, the ethanol was gradually replaced with hexamethyldisilazane (HMDS) using a stepwise exchange (2:1, 1:1, and 1:2 ethanol:HMDS mixtures, 4 min each), followed by immersion in 100% HMDS for 4 min. Excess HMDS was removed, and samples were left to dry completely under a fume hood for at least 16 h.

Prior to imaging, samples were sputter‐coated with a thin conductive gold layer using a JEOL JFC‐1300 Auto Sputter Coater. Samples were mounted onto the SEM sample holder and placed in the sputter chamber. Coating was performed at a current of 20 mA for 30 s employing an argon plasma. SEM imaging was subsequently performed to visualize cell morphology and cell–scaffold interactions at high‐resolution with acceleration voltages ranging from 5 to 20 kV depending on the imaged structure.

### 4.9 Force Analysis in MATLAB

Beam deflection was quantified from confocal microscopy images using a custom semi‐automatic MATLAB script. A single‐plane RGB image of the blue confocal channel was imported, and two user‐selected points at the anchor points of the beam were used to define the baseline orientation. Ninety evenly spaced cross‐sections, oriented orthogonally to the beam longitudinal axis, were then generated along the beam length. The pipeline is shown in Figures S11 and S12. All inputs to the script were performed by a blinded assessor. To improve the reproducibility of the image analysis, the MATLAB workflow was designed to minimize operator input. The user only specifies the beam endpoints and image scale, after which the beam centerline, maximum deflection, and equivalent force location are determined automatically from perpendicular intensity profiles extracted along the beam. A graphical zoom function enables accurate pixel‐level selection of the beam endpoints, thereby reducing operator‐dependent variability (see also Figures S11 and S12). Future work could incorporate artificial intelligence (AI)‐based image analysis and pattern recognition algorithms to automate the analysis of confocal microscopy images, enabling robust real‐time tracking of beam deformation and cellular traction forces.

For each cross‐section, the RGB intensity profile was extracted using linear interpolation. The blue fluorescence channel, which corresponds to the autofluorescence of the beam, was smoothed with a moving‐average filter (window size: 5 pixels) to reduce noise. The blue intensity channel was locally fitted with a second‐order polynomial, and the dominant peak within each profile was identified. The vertex of this polynomial provided the peak location with subpixel precision, yielding the beam centroid for each cross‐section. These centroid positions were transformed into a beam‐aligned coordinate system, resulting in the displacement data. A third‐order polynomial was then fitted to the displacement data to generate a continuous deflection profile. To enforce zero displacement at the anchored ends, the mean offset of the first and last sampling points was subtracted.

The maximum deflection was obtained from the fitted curve. Pixel‐to‐micron conversion was performed by selecting the scale bar, determining its pixel length via binary thresholding, and applying a bounding‐box measurement. All geometric quantities were subsequently converted into physical units. The axial position of maximum bending was extracted from the fitted curve and reported in both pixel and micrometer coordinates relative to the beam origin.

The equivalent point force at the location of maximum bending was calculated using an analytical beam model. Beams with multiple cells or with multiple separate cell attachment points were not included in the analysis. Assuming linear elastic behavior and small deflection theory, the beam stiffness was experimentally derived as described in the section on mechanical characterization.

The equivalent point force was then calculated using $\xi =\frac{a}{L}$ as the normalized position of maximum deflection along the beam (with *a* being the location of the force from the anchor point, *L* the beam length and δ the maximum deflection) via classical Euler–Bernoulli beam theory for a clamped–clamped beam with a point load at an arbitrary position.

(7) $$F\left(\xi \right)=\frac{k_{\mathrm{beam}}}{64\;\xi^{3}{\left(1-\xi \right)}^{3}}\delta$$

All extracted parameters were automatically exported to an Excel file for statistical analysis. The analyzed image with overlaid deflection curve and annotated maximum displacement was also automatically saved. The derivation is provided in Figure S17. The data were analyzed with another MATLAB script, linking the data to the respective wells, averaging the deflection and forces, and generating the boxplots.

### 4.10 Statistical Analysis

All analyses were performed in MATLAB. Linear mixed‐effects models were used to account for repeated measurements within patient cell lines and samples, with group (CNTRL vs. TSC2) as a fixed effect and cell line as a random effect, including time point as an additional fixed effect. Model assumptions were assessed by residual inspection and Lilliefors tests. As force data deviated from normality, a log transformation was applied. Both raw and log‐transformed models were evaluated. To avoid pseudoreplication, analyses were repeated on per‐patient averages using one‐way ANOVA, and nonparametric comparisons were performed using the Mann–Whitney *U*‐test. All methods yielded consistent results, with no significant differences between groups. Statistical significance was defined as *p* < 0.05. In addition to *p*‐values, effect sizes (Cohen's *d*) and corresponding 95% confidence intervals were calculated for all major group comparisons to provide estimates of the magnitude and precision of the observed effects.

## Author Contributions

**Pieter van Altena**: Conceptualization, Methodology, Software, Data curation, Investigation, Validation, Formal analysis, Visualization, Writing – original draft. **Lucia Castillo Ransanz**: writing – review and editing, methodology, investigation, and validation. **Ruben H. Guis**: methodology and writing – review and editing. **Giulia Bergamaschi**: methodology. **Nanne J. Paauw**: methodology. **Urs Staufer**: methodology and writing – review and editing. **Vivi M. Heine**: supervision, funding acquisition, project administration, resources, writing – review and editing, and investigation. **Angelo Accardo**: conceptualization, methodology, investigation, validation, supervision, funding acquisition, project administration, resources, and writing – review and editing.

## Funding

This research was funded by the NWO (Nederlandse Organisatie voor Wetenschappelijk Onderzoek) ENW‐M‐2 grant number OCENW.M20.047.

## Ethics Statement

The use of human cells in this study was reviewed and approved by the Institutional Review Board (IRB) of Amsterdam UMC. TSC patient‐derived cells were obtained from the Coriell Institute for Medical Research under a Material Transfer Agreement (MTA). All human‐derived materials, including those collected at Amsterdam UMC, were obtained with appropriate informed consent and ethical approval in accordance with applicable regulations.

## Conflicts of Interest

The authors declare no conflicts of interest.

## Supporting information




**Supporting File 1**: adma74596‐sup‐0001‐SuppMat.pdf.

**Supporting File 2**: adma74596‐sup‐0002‐MovieS1.mp4.

**Supporting File 3**: adma74596‐sup‐0003‐MovieS2.mp4.

**Supporting File 4**: adma74596‐sup‐0004‐MovieS3.mp4.

**Supporting File 5**: adma74596‐sup‐0005‐MovieS4.mp4.

**Supporting File 6**: adma74596‐sup‐0006‐MovieS5.mp4.

**Supporting File 7**: adma74596‐sup‐0007‐MovieS6.mp4.

**Supporting File 8**: adma74596‐sup‐0008‐MovieS7.mp4.

**Supporting File 9**: adma74596‐sup‐0009‐MovieS8.mp4.

## Data Availability

All details needed for repeating the experiments are provided in the experimental section. The data that support the findings of this study are available from the corresponding author upon reasonable request.
