# Supplementary material for: Elastomeric 3D‐Printed Microenvironments Enable Nanonewton Force Measurements in Healthy and Diseased Human Pluripotent Stem Cell‐Derived Neuroepithelial Cells
Source: Adv Mater. 2026 Aug 17;38(56):e74596. doi: 10.1002/adma.74596 (PMC13648881; doi:10.1002/adma.74596)
Supplement: Supplementary file 1 [file ADMA-38-e74596-s004.pdf]

Supplementary information

Elastomeric 3D-printed microenvironments enable nanonewton force measurements in healthy and diseased human pluripotent stem cell-derived neuroepithelial cells

Pieter F.J. van Altena [a,†], Lucia Castillo Ransanz [b,†], Ruben H. Guis [a], Giulia Bergamaschi[c], Nanne J. Paauw [c], Urs Staufer[a], Vivi M. Heine [b,d*], Angelo Accardo[a,*]

[a]Department of Precision and Microsystems Engineering, Faculty of Mechanical Engineering, Delft University of Technology, Mekelweg 2, 2628 CD Delft, The Netherlands.

[b]Department of Child and Adolescence Psychiatry, Emma Center for Personalized Medicine, Amsterdam UMC, De Boelelaan 1100, Amsterdam, 1081 HZ, The Netherlands.

[c]Microscopy and Cytometry Core Facility, Department of Molecular Cell Biology and Immunology, Amsterdam UMC location Vrije Universiteit Amsterdam, Amsterdam, the Netherlands

[d]Department of Complex Trait Genetics, Centre for Neurogenomics and Cognitive Research, Amsterdam Neuroscience, Vrije Universiteit Amsterdam, Amsterdam, the Netherlands

[†]*Equal contribution*

**Corresponding authors' e-mail:* A.Accardo@tudelft.nl, VM.Heine@amsterdamumc.nl,

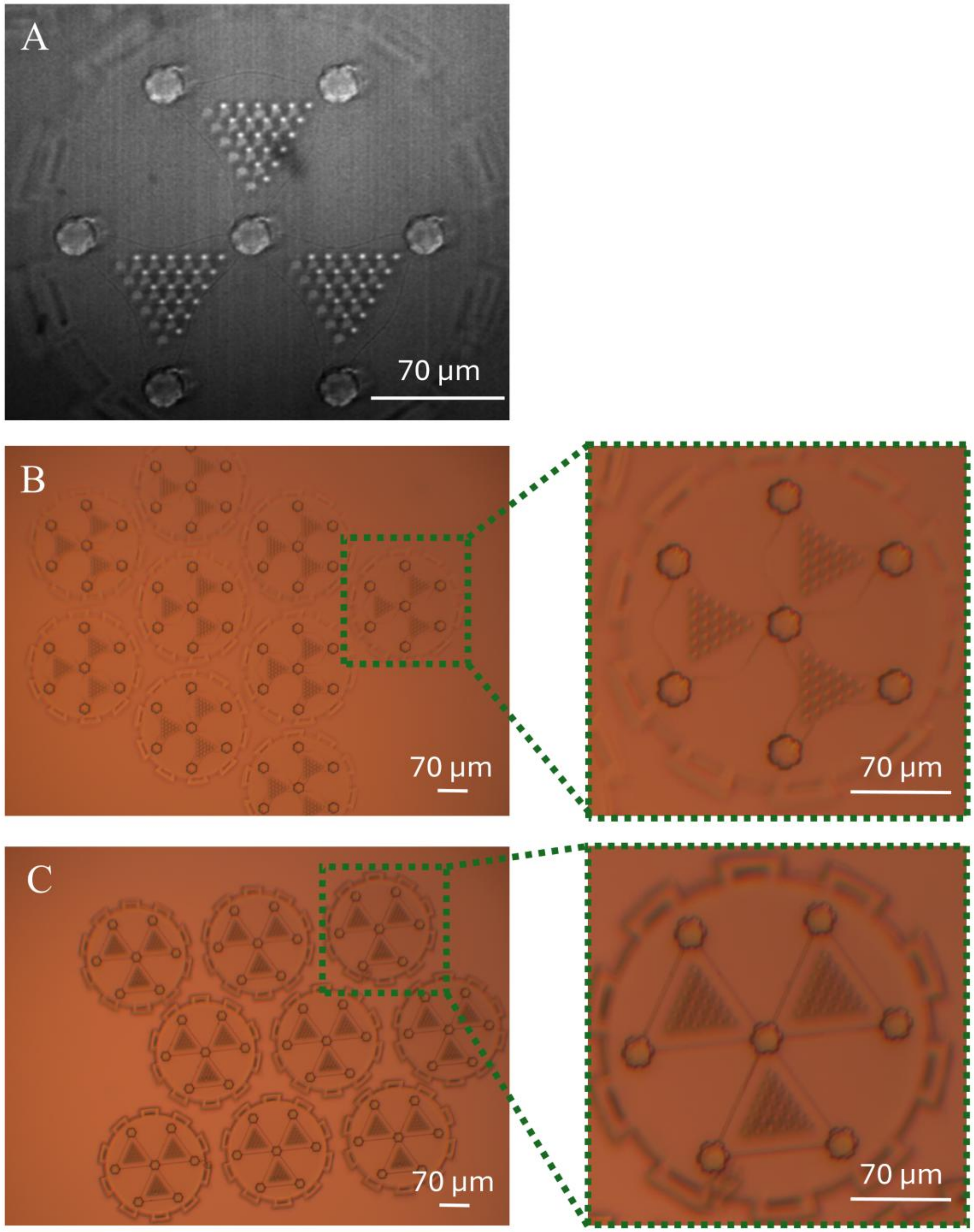

Figure S1: ***Beam curvature during fabrication and post-processing.*** *(A) In situ view during printing, showing visibly curved beams due to swelling effects. (B) Structures immersed in isopropanol (IPA), where beam curvature persists as a result of solvent-induced swelling. Insets highlight individual units with bent beams. (C) After rinsing with NOVEC 7100 and drying, the beams recover a straight geometry, indicating removal of swelling-induced deformation. Insets show the restored straight-beam configuration. Scale bars: 70 µm.*

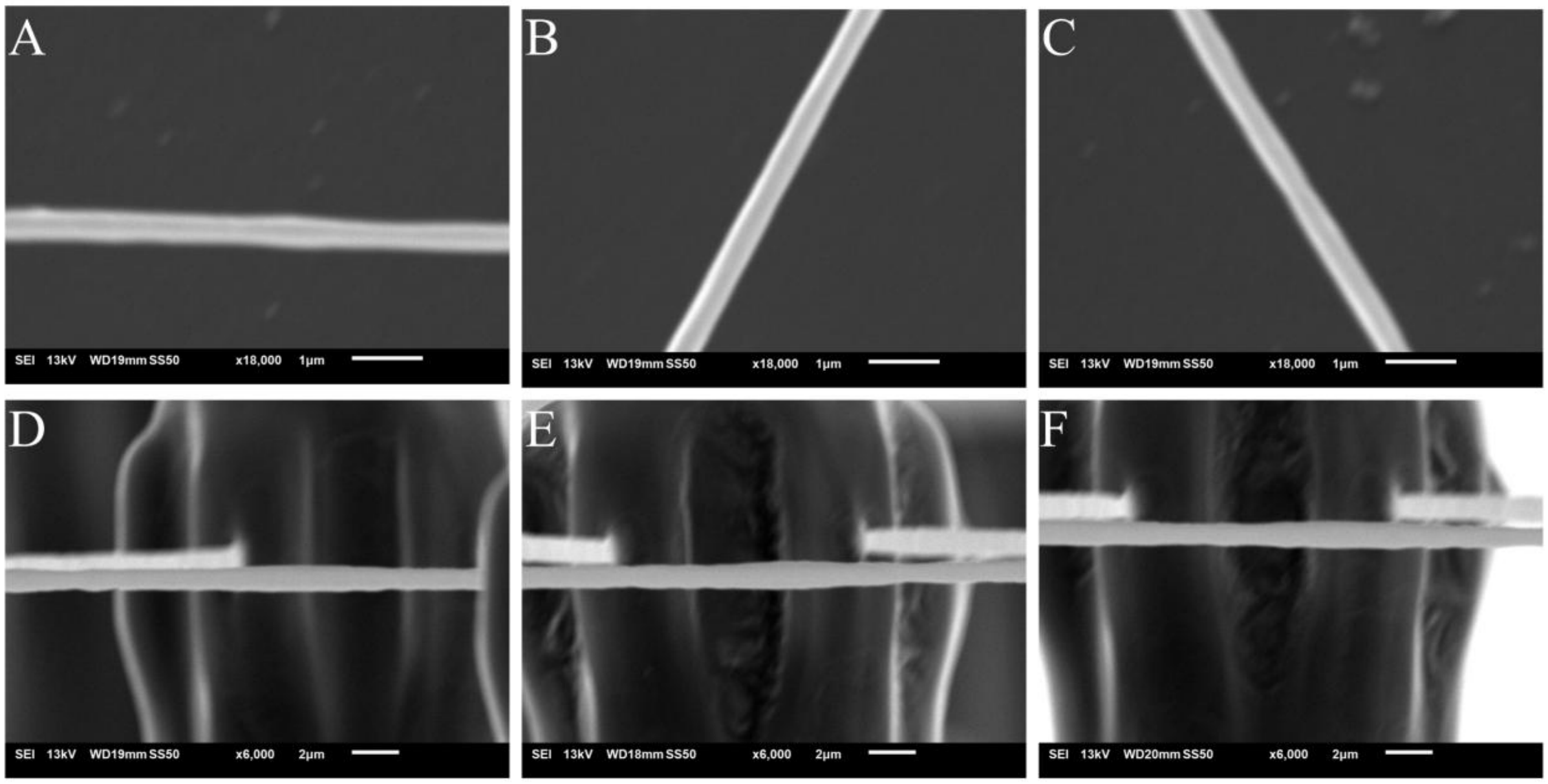

Figure S2: ***SEM characterization of minimum feature size achieved at high TBAF concentration (0.88 M) for 75 seconds.*** *(A,B,C) SEM image of minimum achieved features in the XY plane down to 432 ± 11 nm (n = 3). (D,E,F) SEM image of minimum achieved features in the Z plane down to 1017 ± 70 nm (n = 3). These images demonstrate the possibilities of the method under high-concentration etching conditions.*

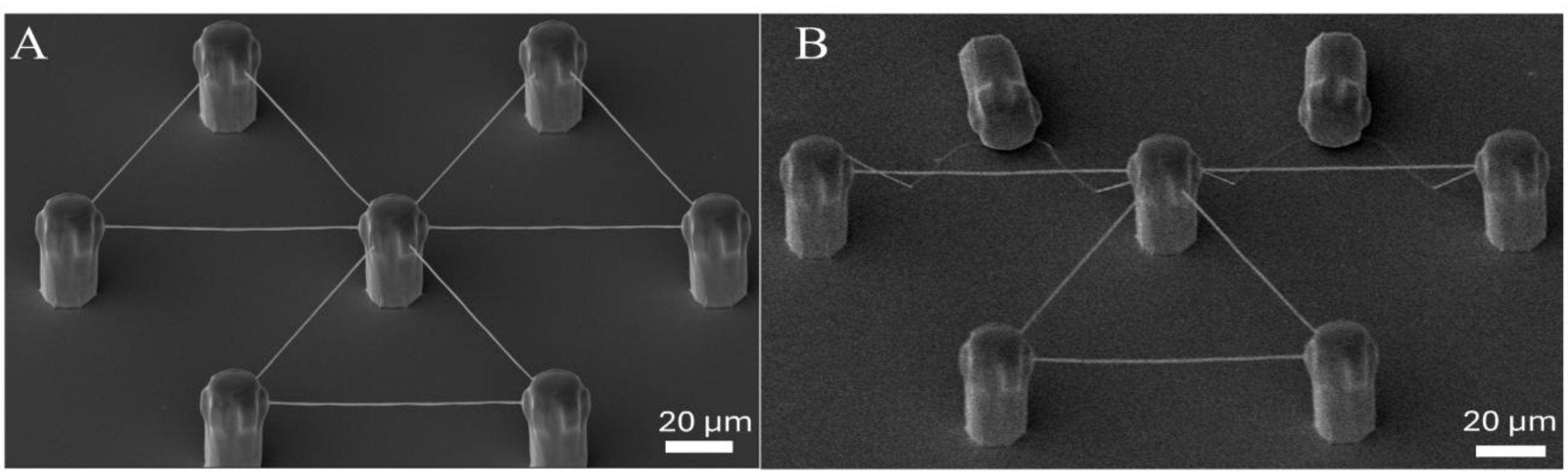

Figure S3: ***Mechanical instability induced by excessive thinning during etching for same etch exposures.*** *(A) Intact structure after etching, maintaining mechanical integrity with suspended beams. Structure after 60 s etching at high concentration. (B) Structure after 60 s etching at high concentration, showing significant collapse and partial detachment due to extreme thinning and reduced mechanical stability during drying. Scale bars: 20 µm.*

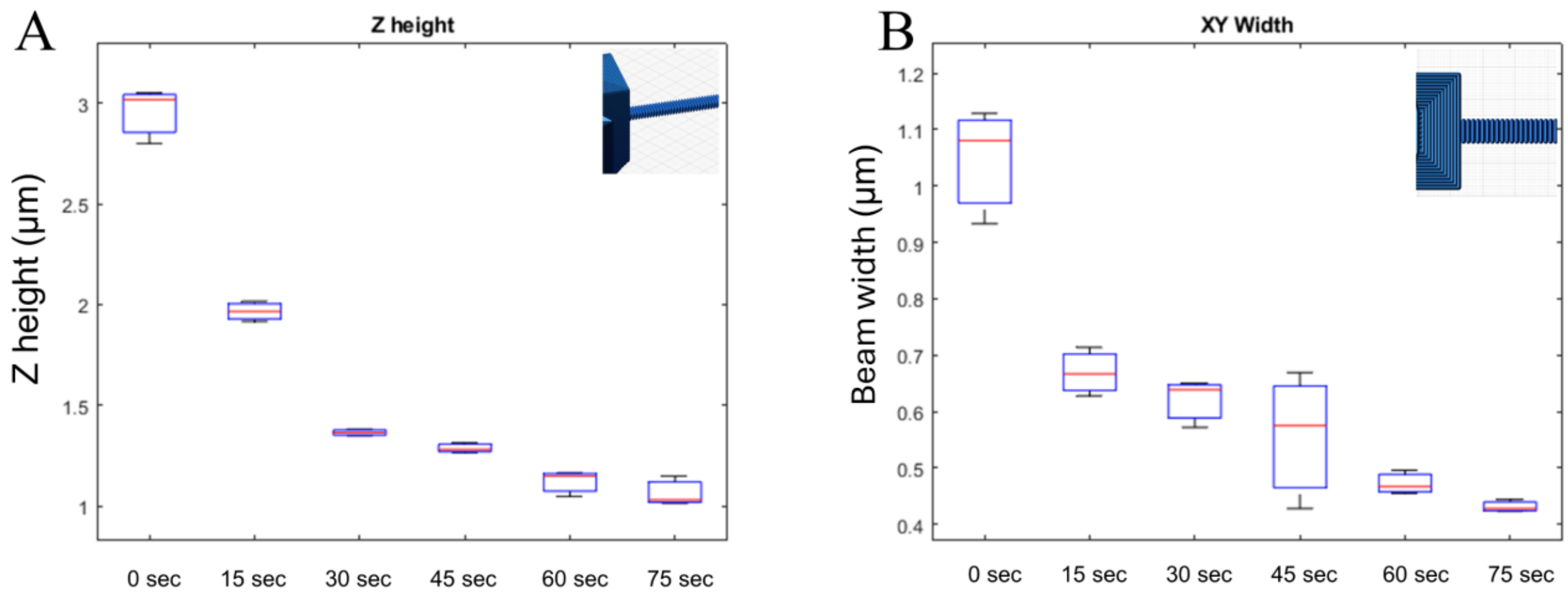

Figure S4: ***Evolution of beam dimensions with etching time at high TBAF concentration (0.88M).***
(A) Z-height of the beams as a function of etching time (0–75 s), showing a progressive decrease in height with increasing exposure. (B) Corresponding beam width in the XY plane, also decreasing with etching time, with increased variability at intermediate times due to non-uniform thinning. Insets illustrate representative beam cross-sections. Overall, prolonged exposure to the etchant leads to significant dimensional reduction, but excessive etching compromises structural integrity, consistent with the collapse observed in Supplementary Figure S3. Error bars represent standard deviation (n = 7). Test performed on single beams supported by two pillars.

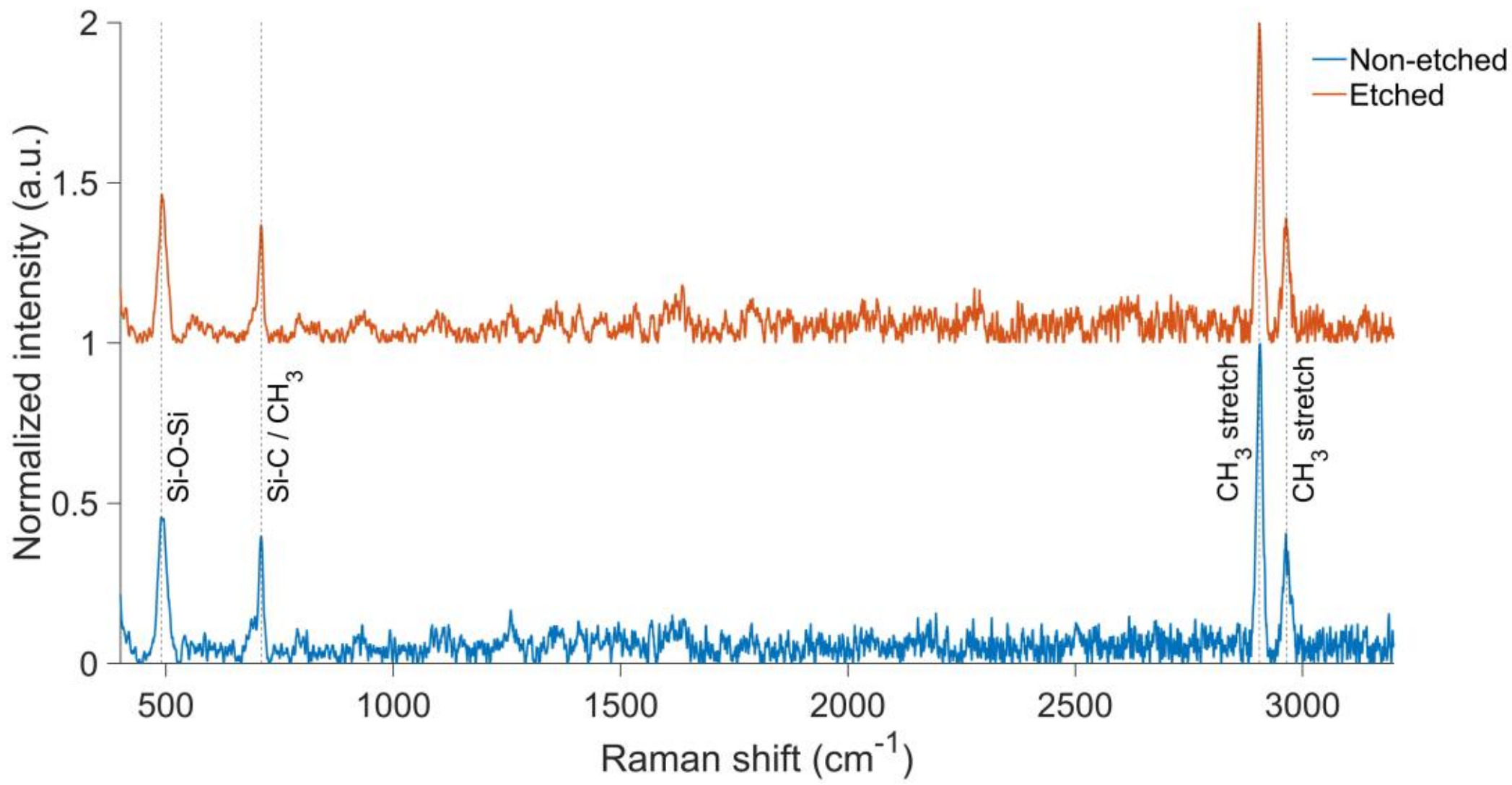

Figure S5: ***Raman spectra of IP-PDMS structures before and after etching****. The non-etched sample (blue) and etched sample (orange) are shown after normalization, enabling direct comparison of characteristic vibrational modes. Prominent peaks corresponding to Si–O–Si bending/symmetric stretching (~490 cm^{-1}), Si–CH_3 (~710 cm^{-1}), and C–H_3 symmetric and asymmetric stretching vibrations (~2900–3000 cm^{-1}) are indicated.*

Raman spectroscopy was employed to assess whether the wet etching process altered the chemical composition of the IP-PDMS microstructures. Measurements were performed on both etched and non-etched samples using a confocal Raman microscope under ambient conditions. Spectra were collected from representative regions of the printed structures to evaluate potential chemical modifications introduced during the TBAF etching process. The Raman spectra of both etched and non-etched samples exhibited the characteristic vibrational bands of PDMS, including the Si–O–Si stretching modes, CH_3 deformation modes, and Si–CH_3 vibrations. Comparison of the spectra showed no significant shifts in peak positions or the appearance of additional peaks after etching, indicating that the chemical backbone of the IP-PDMS polymer remained largely intact (shown in Figure S5). Minor variations in peak intensity were attributed to slight differences in surface morphology and measurement location rather than chemical changes.

These results confirm that the TBAF-based wet etching process primarily modifies the geometry of the printed structures by reducing the cross-sectional dimensions of the beams, while preserving the fundamental chemical structure of the polymer. Consequently, the etching process enhances mechanical sensitivity without introducing detectable chemical degradation of the material.

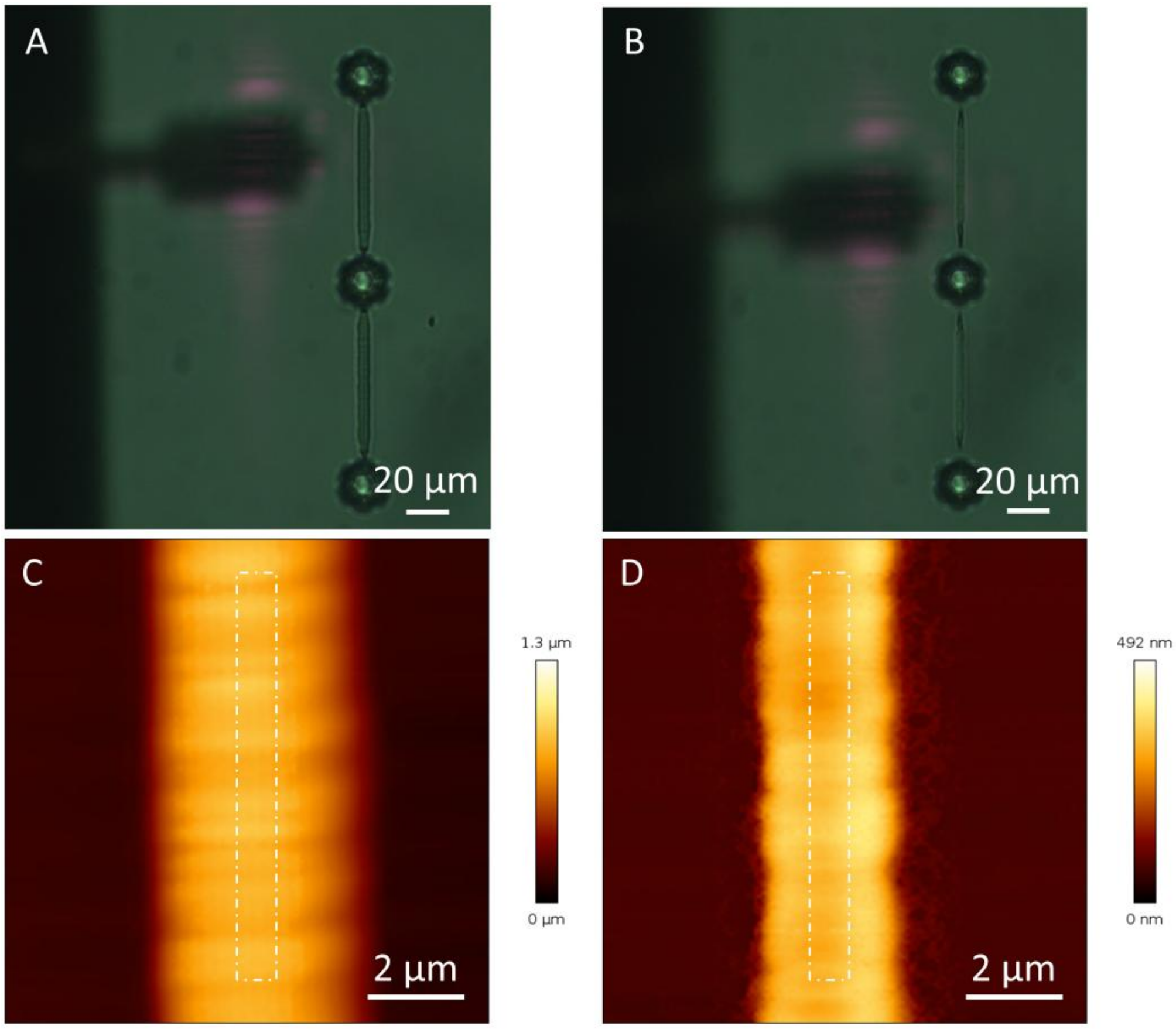

Figure S6. ***AFM comparison of IP-PDMS beam surfaces before and after TBAF etching.*** *Optical/AFM overview images of non-etched (A) and etched (B) beams and corresponding AFM height maps of the beam surface before (C) and after (D) etching. Roughness was quantified within the white dashed regions along the center of the beam. The average Ra values were 20.37 ± 5.996 nm for non-etched beams and 18.64 ± 1.998 nm for etched beams.*

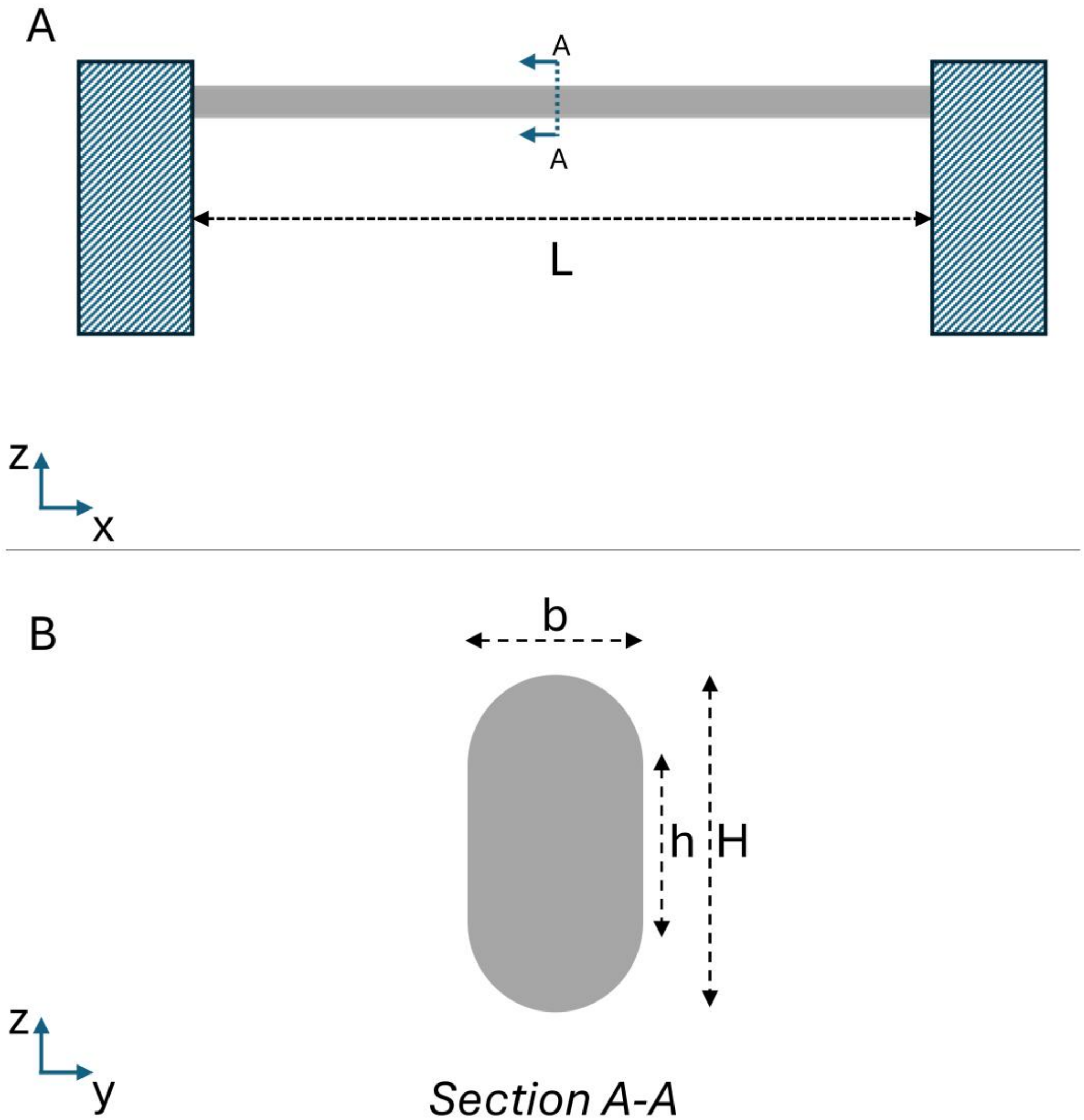

Figure S7 ***Schematic of the double-clamped beam geometry and cross-section used for mechanical modeling.*** *(A) Side view of a suspended double-clamped beam of length L, fixed at both ends. Section A–A marks the location of the cross-sectional view shown in B. (B) Cross-section (A–A) of the beam, showing the characteristic geometry with width b and height H. This geometry is used to estimate the cross-sectional moment of inertia (I) and to model the maximum deflection of the beam under a central point load using Euler–Bernoulli beam theory with the force perpendicular to the beam in the direction of b.*

A

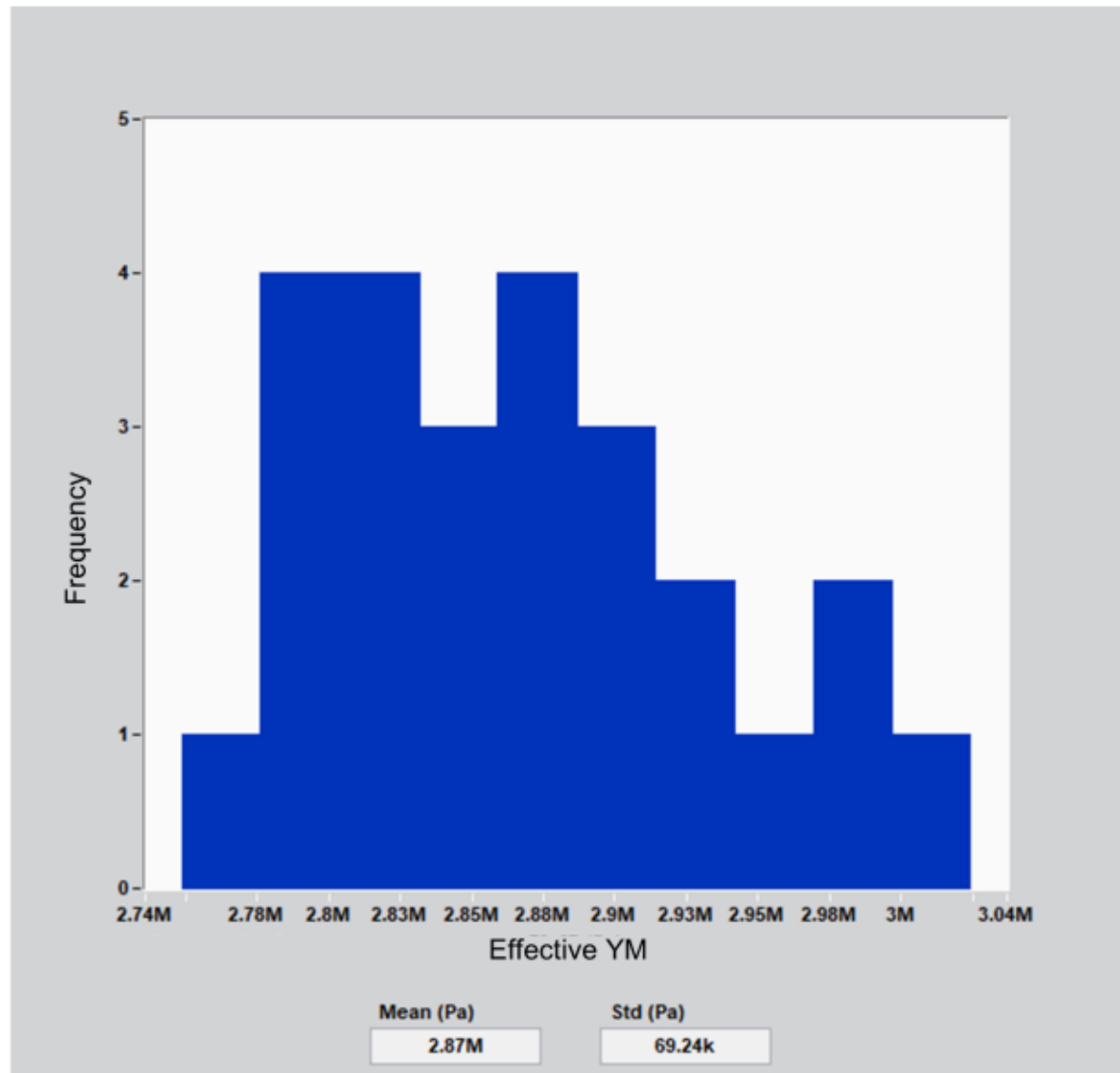

B

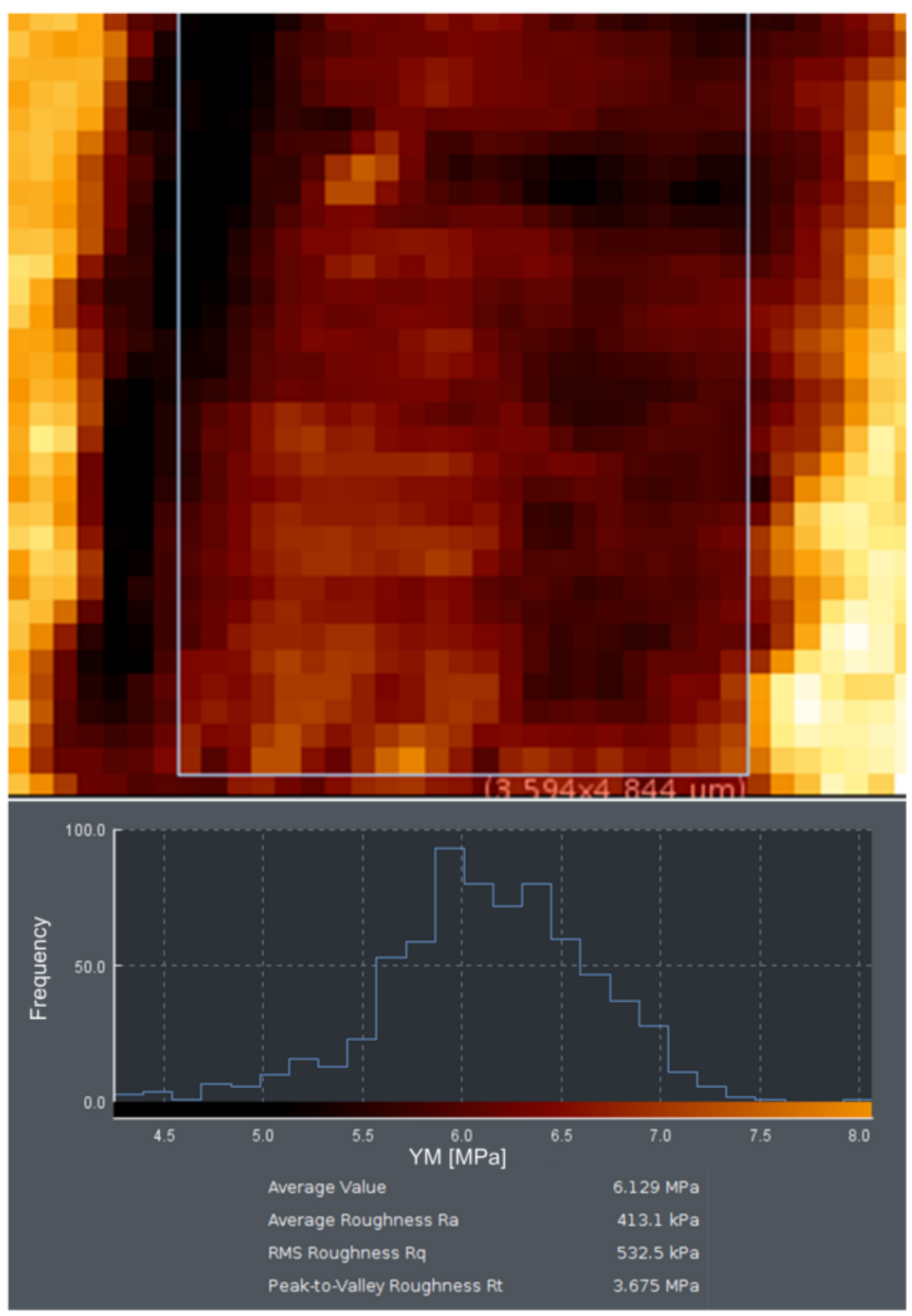

Figure S8: ***Mechanical characterization.*** *Nanoindentation results showing an average Young's modulus of ~2.9 MPa obtained from a matrix scan comprising 25 measurements. (B) AFM-based mechanical mapping indicating a higher average Young's modulus of ~6.1 MPa obtained from 713 measurements, alongside corresponding surface topography and roughness analysis.*

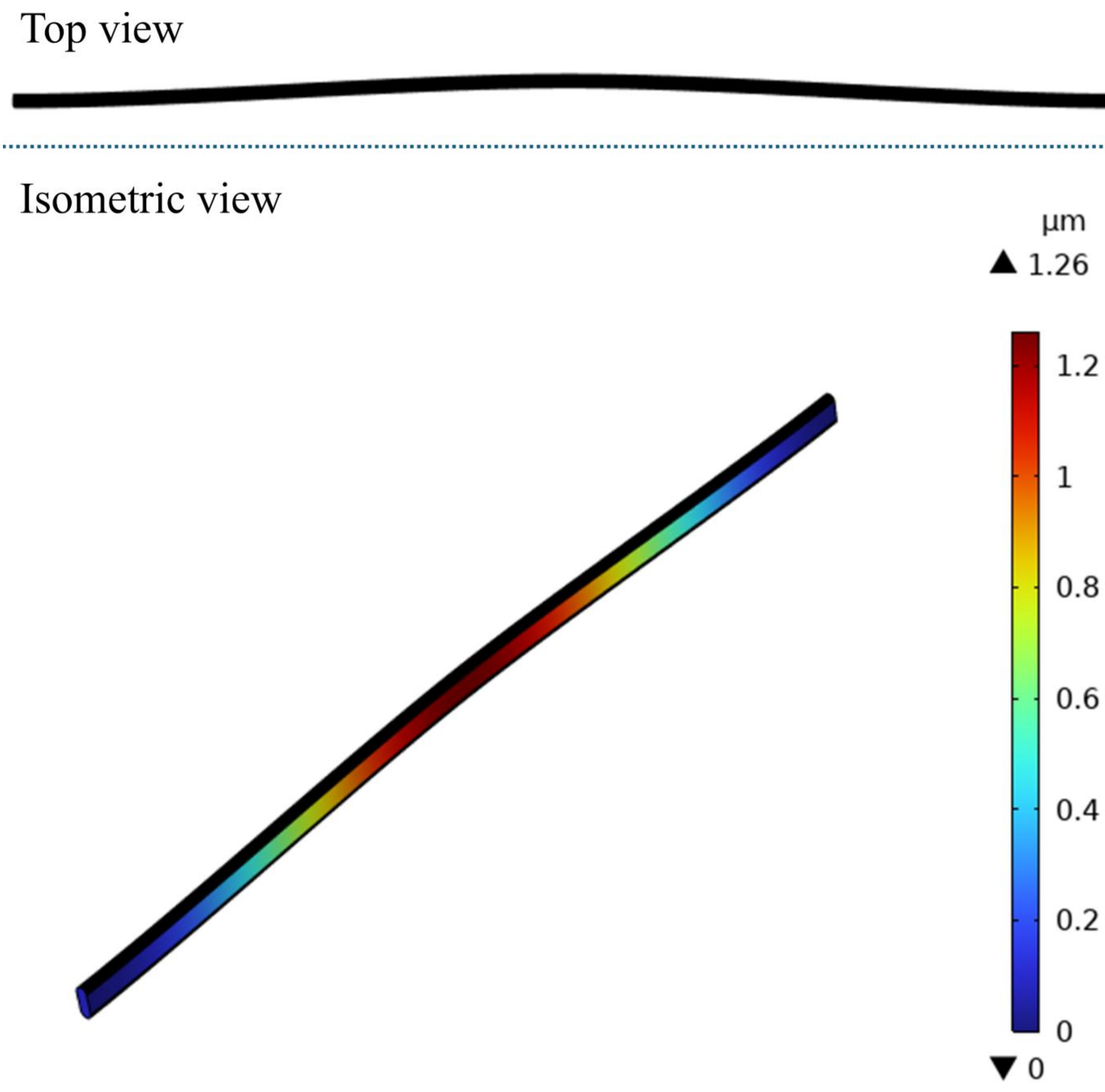

Figure S9: ***Finite element simulation of beam deflection performed in COMSOL Multiphysics****. A) simulated top view of the deformed beam profile. B) isometric view showing the three-dimensional deformation with a displacement map. The color scale represents the magnitude of displacement (µm), with maximum deflection occurring at the beam center and minimal displacement at the clamped boundaries, consistent with the behavior of a doubly clamped beam under transverse loading. Simulation for 1 nN loading in the center of the beam.*

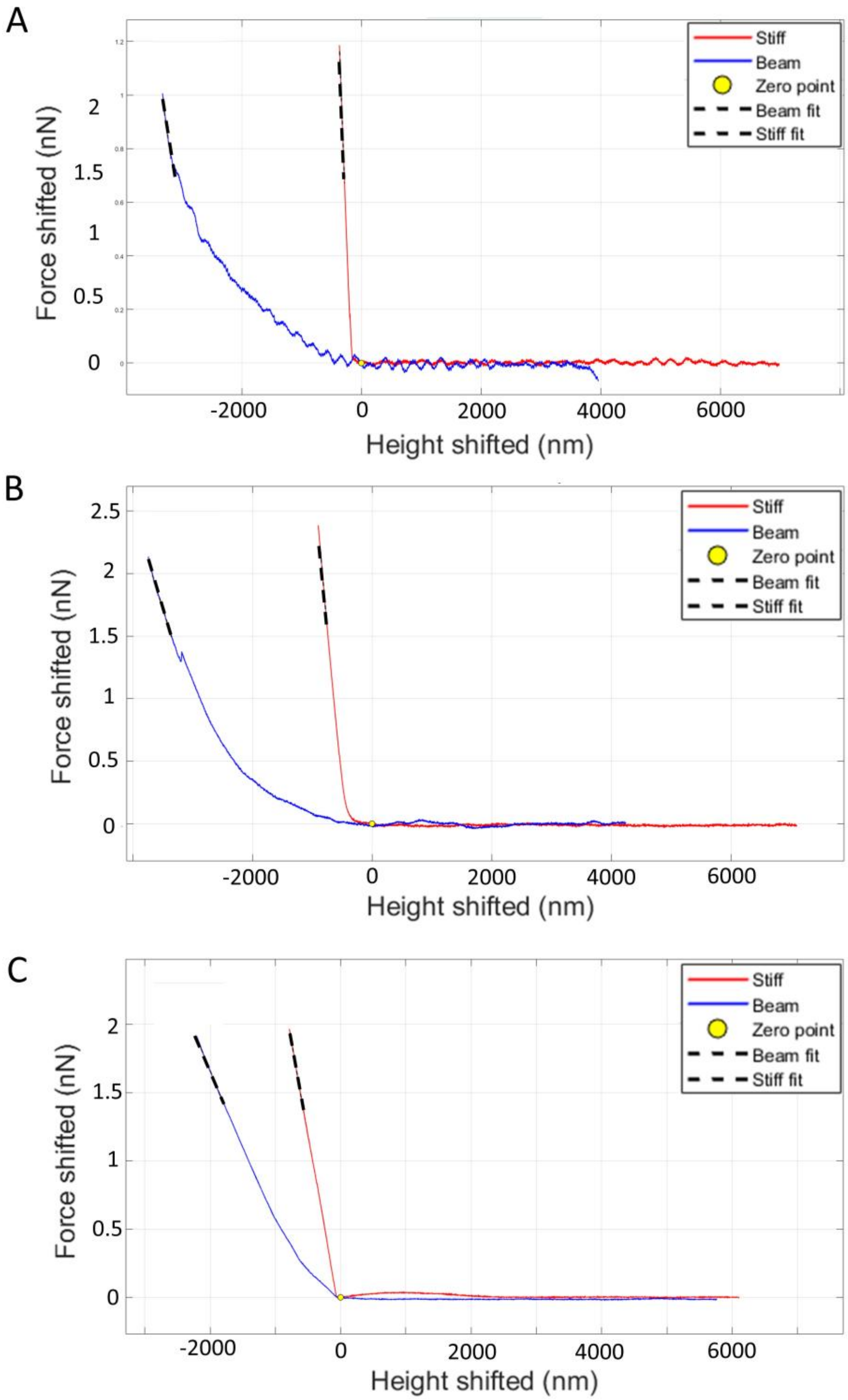

Figure S10: ***AFM stiffness characterization*** *of IP-PDMS beams after incubation in physiological imaging medium (Invitrogen, A59688DJ) at 37 °C. Representative force–displacement curves acquired after* ***(A) 1 h (T1)****,* ***(B) 24 h (T2)****, and* ***(C) 53 h (T3)*** *of incubation. The beam stiffness was determined from the linear structural response region (dashed black lines). All force curves were corrected for cantilever sensitivity. The extracted beam stiffness values remained stable over time, indicating no measurable degradation of the structural spring constant during prolonged exposure to medium.*

1) Load blue channel Z projection

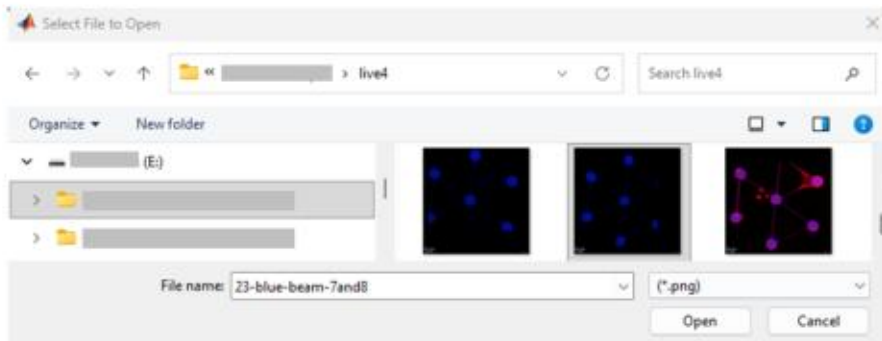

2) Select endpoints of beam

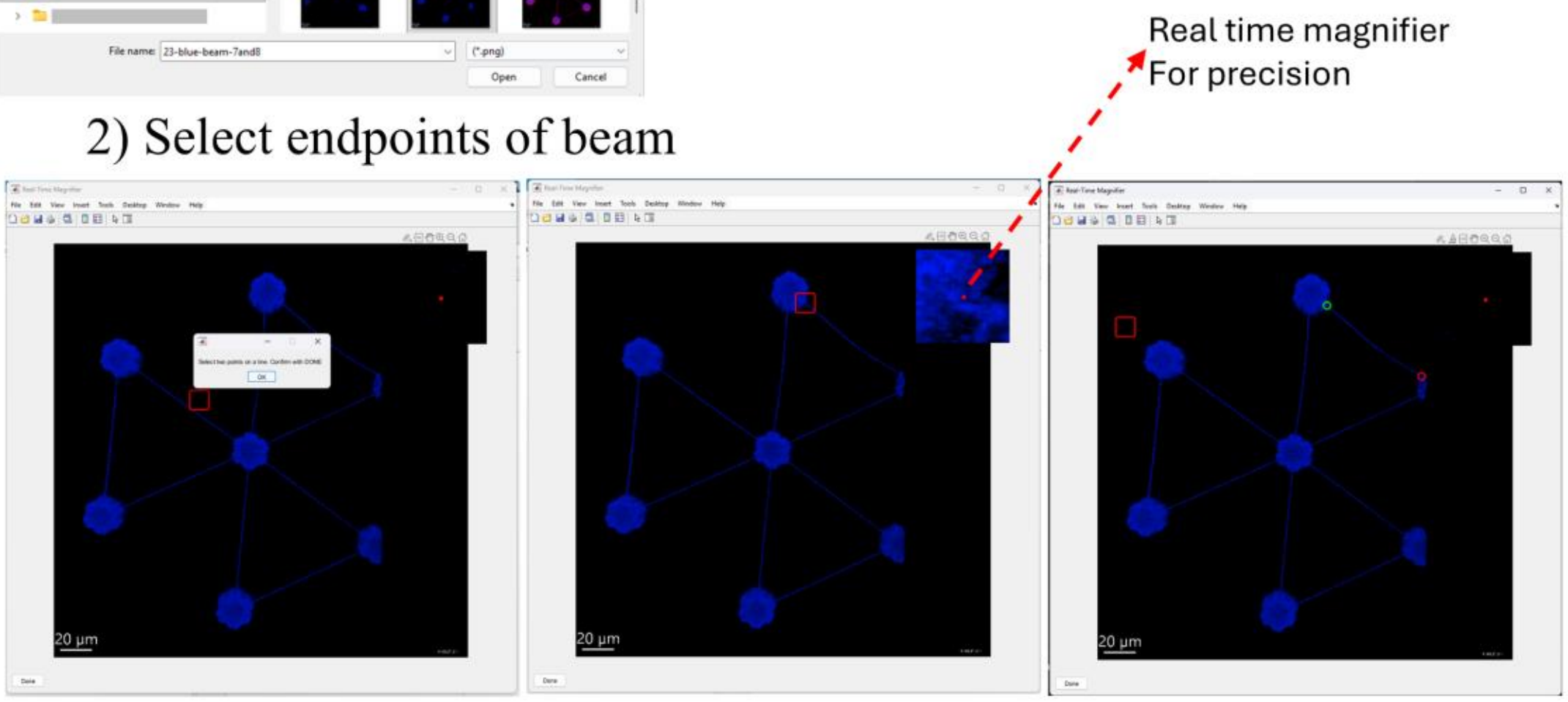

3) Perpendicular lines and intensity profiles plotted

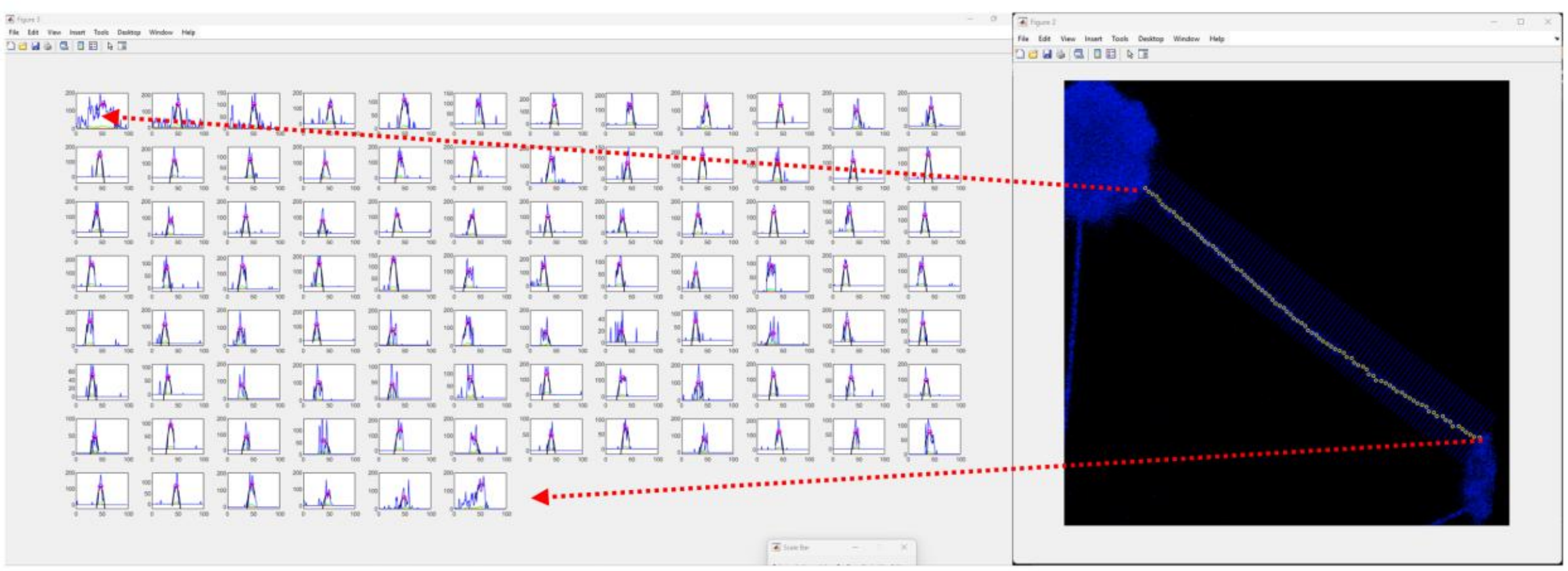

4) Select scalebar

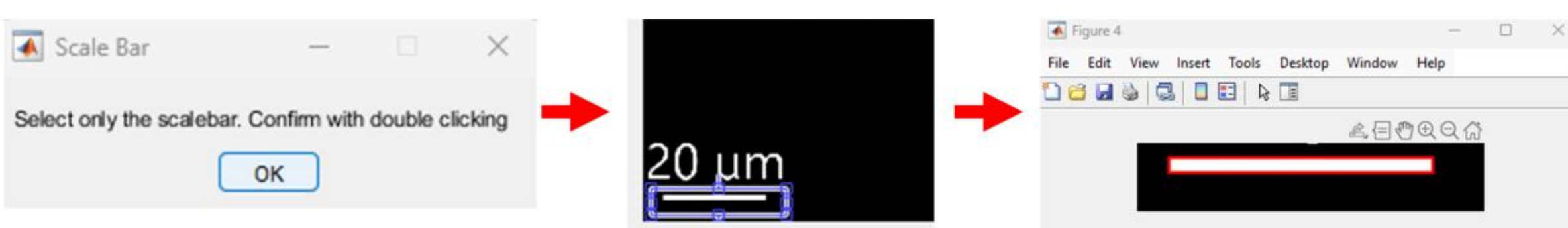

5) Input scalebar information

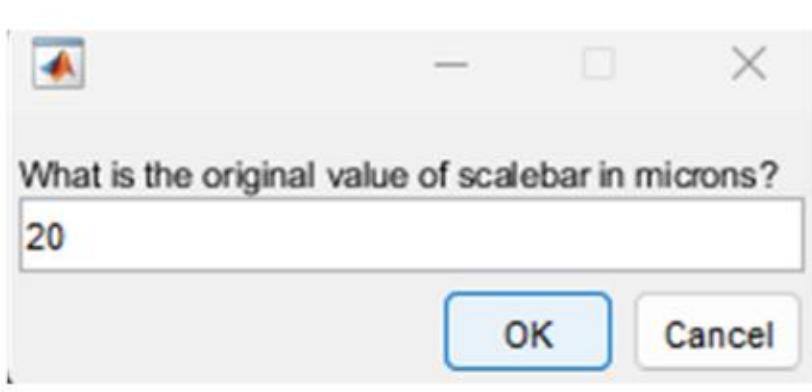

6) Automated output

Figures

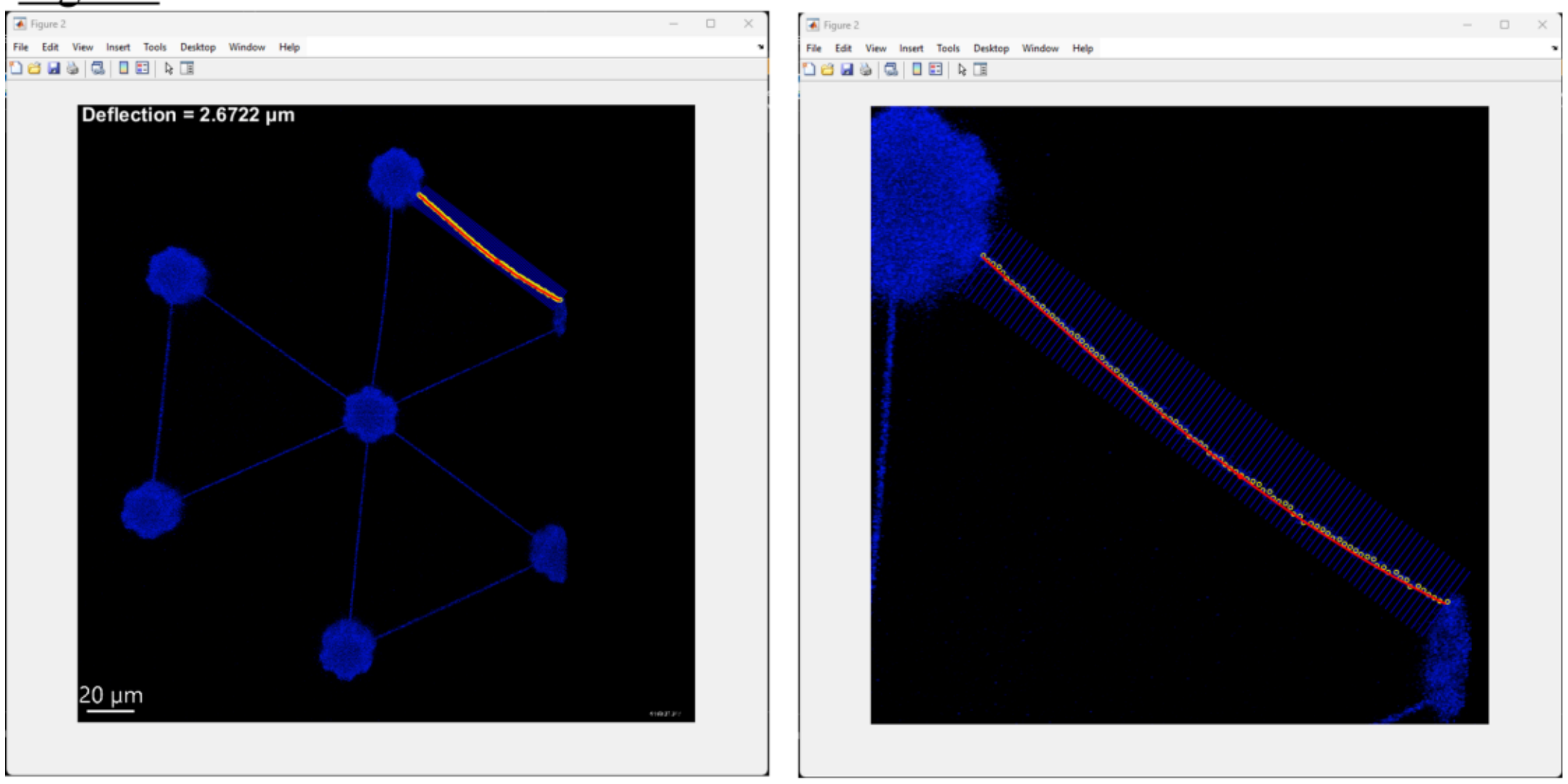

Quick check data

Command Window

```
Clicked at IMAGE coordinates: 1659, 439
Clicked at IMAGE coordinates: 2343, 951
First selected point: 1659, 439
Second selected point: 2343, 951
Scalebar length (pixels): 231
           sse: 377.6094
       rsquare: 0.9564
           dfe: 86
    adjrsquare: 0.9549
          rmse: 2.0954

Total beam length: 854.4004 pixels
Total beam length: 73.9741 microns
Maximum bending occurs at 43.2208 microns from the beam origin (pt1).
(In pixels: 499.2002)
Saved analysed image: E:\backup Data paper and MATLAB\Confocal analysis\Results Analysed\23-blue-beam-7and8-ana.png
Equivalent point force at max bending position: 2.914 nN

myFolders =

  5×1 cell array

    {'E:'                         }
    {'backup Data paper and MATLAB'}
    {'Confocal analysis'          }
    {'Results Analysed'           }
    {'23-blue-beam-7and8-ana.png'  }

Results saved to results.xlsx
fx >>
```

Saved data for analysis (Excel)

	A	B	C	D	E	F
1	SavedImageName	MaxBendingMicron	MaxBendingLocationMicron	BeamLengthMicron	RSquared	F_nN
2	23-blue-beam-7an(	2,672239434	43,22079817	73,9740584	0,956407	2,914

Figure S11: ***MATLAB pipeline***

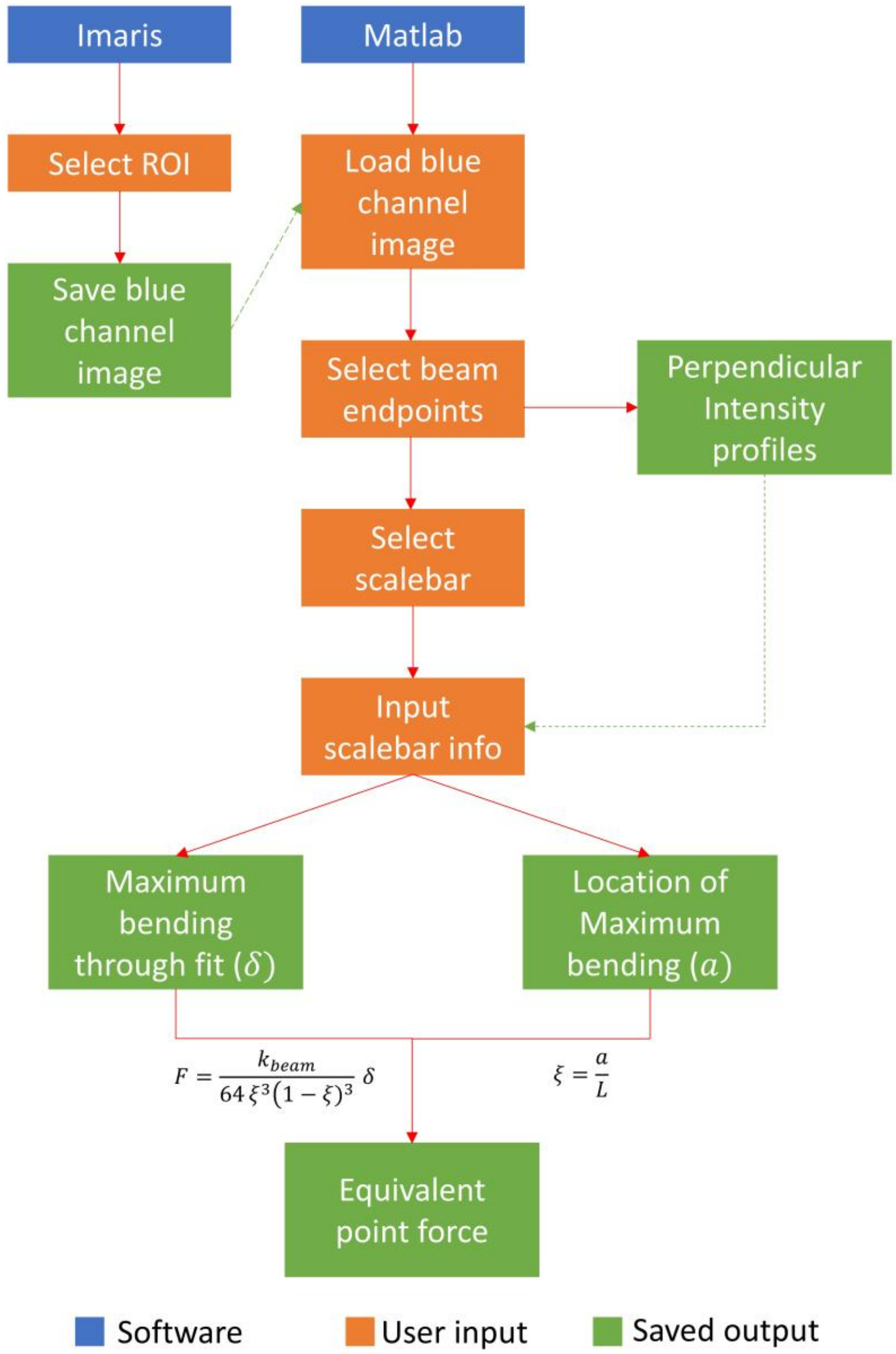

Figure S12. ***Workflow of the image analysis and force calculation pipeline used to quantify cellular traction forces from beam deflection****. Confocal image stacks were first processed in Imaris to define the region of interest (ROI) and export the blue fluorescence channel containing the microcantilever. The exported image was subsequently analyzed in MATLAB, where the user selected the beam endpoints and image scale. Perpendicular intensity profiles were automatically extracted along the beam to determine the beam centerline, from which the maximum beam deflection (δ) and its location (a) were obtained. The normalized force application position was calculated as ξ = a/L, where L is the beam length. Finally, the equivalent point force was calculated using the analytical beam model based on the measured deflection, beam stiffness (k_{beam}), and the position of the equivalent force. Blue boxes indicate software modules, orange boxes represent user inputs, and green boxes denote generated outputs. The MATLAB workflow is shown in supplementary Figure S11 and the derivations of the equations can be found in supplementary Figure S17.*

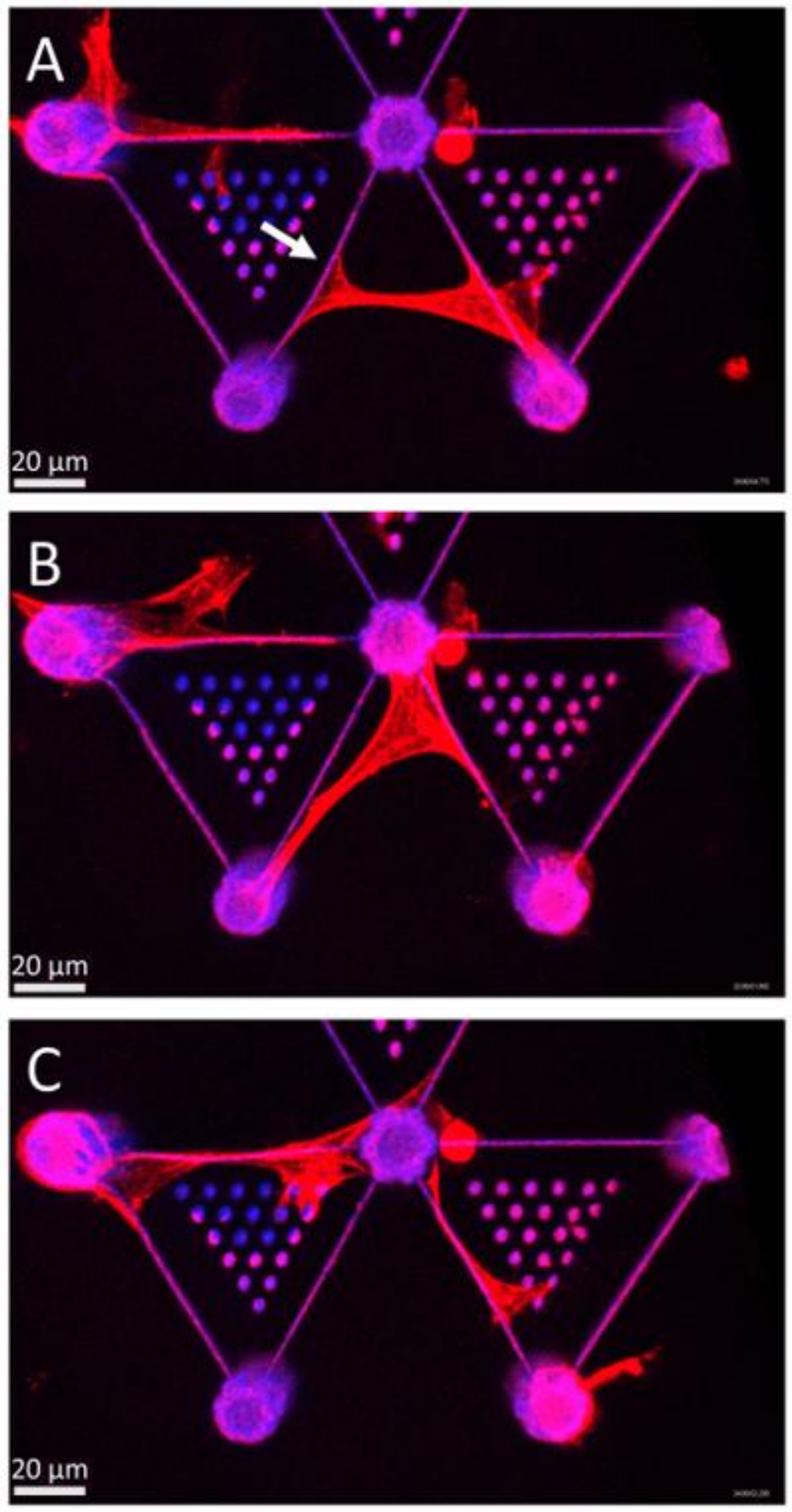

Figure S13: ***Time-lapse fluorescence microscopy showing the elastic recoil of the beams following cell migration****. (A) A cell (F-actin, red) is attached to and exerts traction forces on the beams, resulting in beam deflection indicated with an arrow. (B) As the cell migrates away, the cantilevers begin to recover toward their undeformed position. (C) Following cell detachment, the cantilevers exhibit further elastic recoil, returning to the original configuration. This reversible deformation demonstrates that the platform can dynamically resolve both the application and release of cellular traction forces over time. Scale bars = 20 µm.*

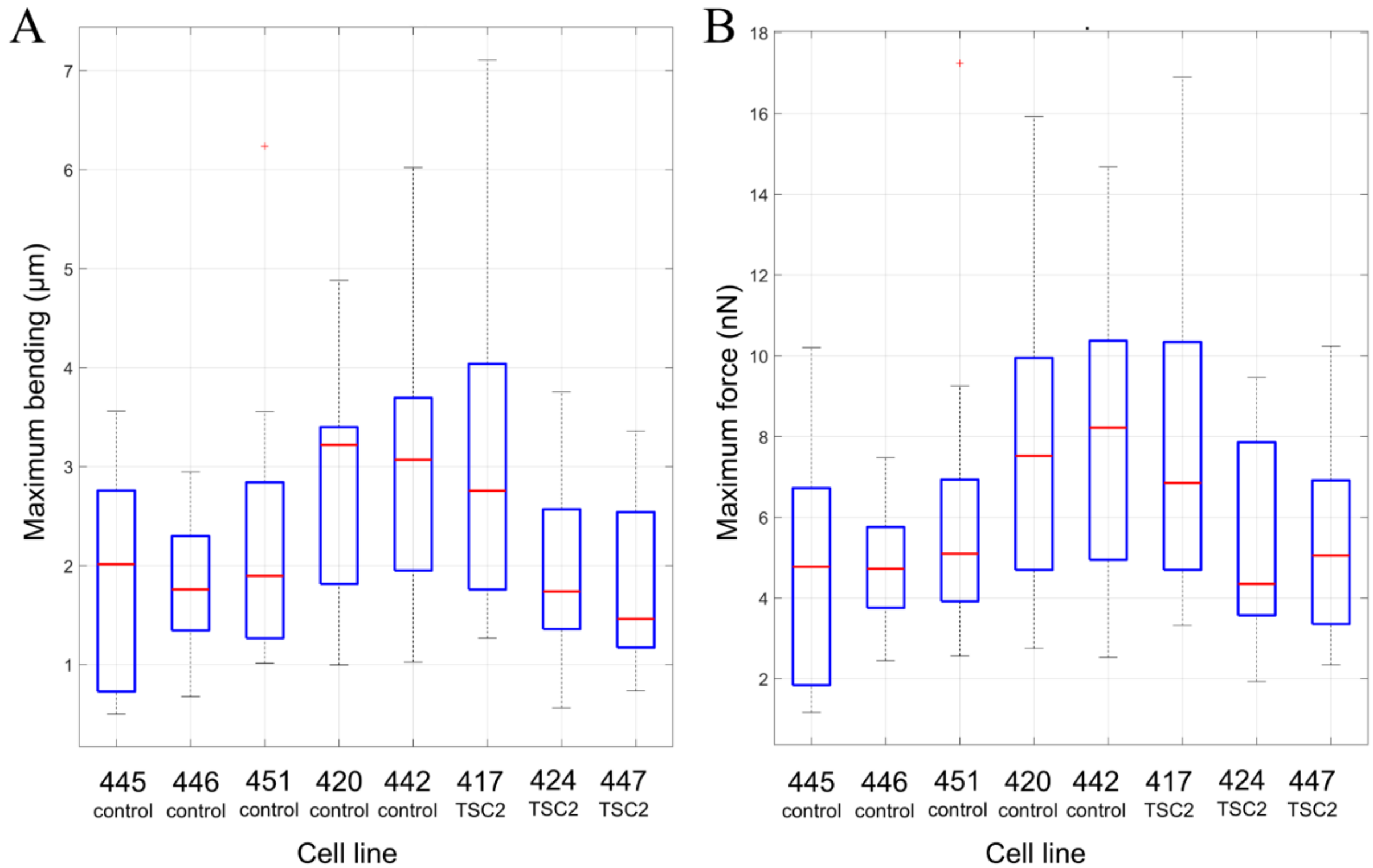

Figure S14: **Beam deformation (A) and force plots (B) per cell line**.

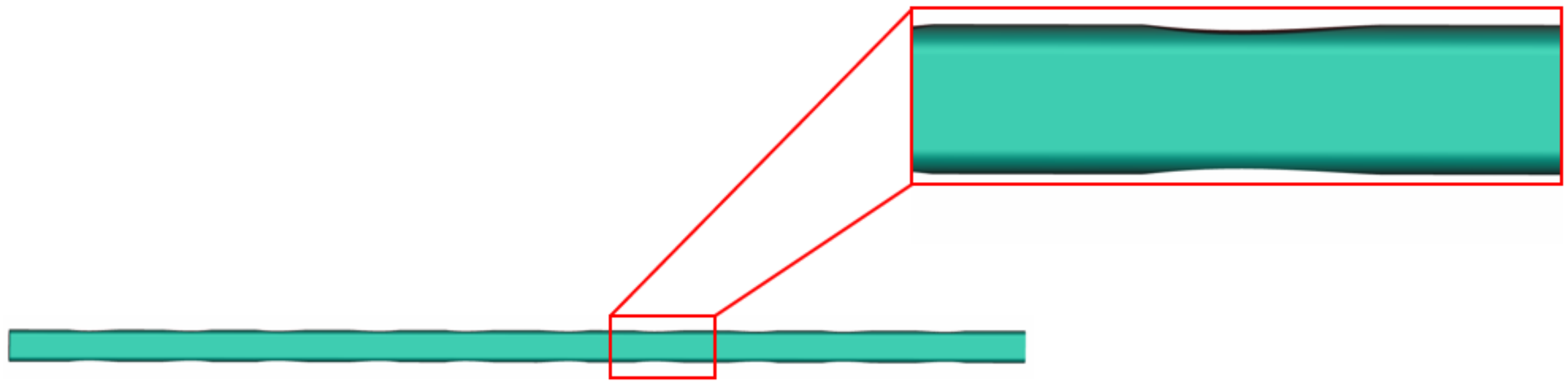

Figure S15: ***Non-uniform beam geometry used in the FEM simulations****. The magnified inset highlights the imposed geometric imperfections, modeled as surface deviations of approximately 120 nm on both sides of the beam. These deviations exceed the typical observed variations and were included as a conservative worst-case scenario to assess the sensitivity of beam deflection to fabrication-induced geometric non-uniformities.*

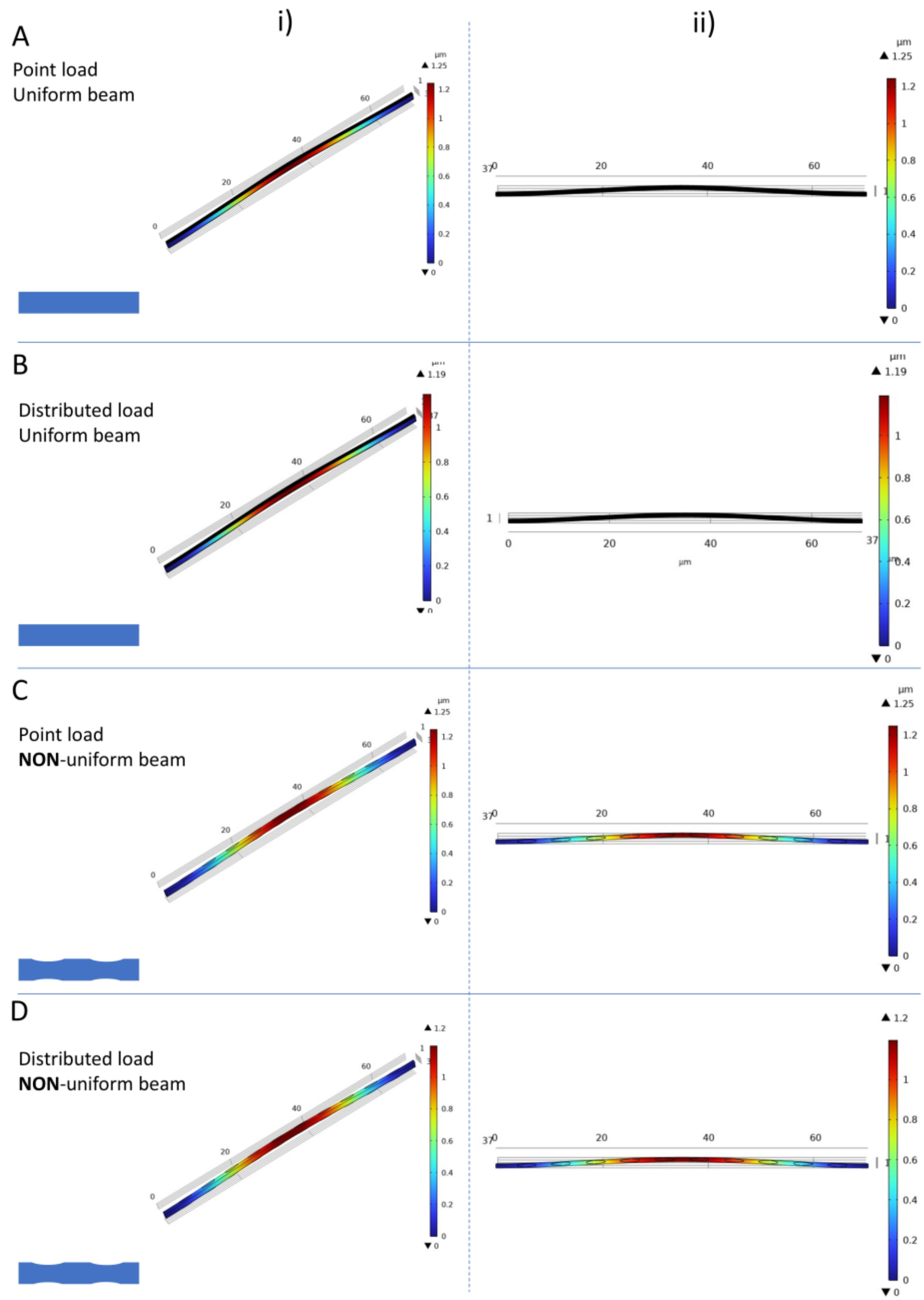

Figure S16: ***FEM comparison of concentrated point-load and uniformly distributed-load assumptions for a 70 µm-long IP-PDMS beam****. Simulations were performed for both an idealized uniform beam geometry (A and B) and a non-uniform beam geometry incorporating lateral imperfections of approximately 120 nm per side (C and D). The distributed load was applied uniformly over a 20 µm region corresponding to the experimentally observed cell–beam adhesion length. i): isometric view of beam deformation. ii) top view of beam deformation. The predicted maximum deflections were 1.25 µm for the point-load cases and 1.19–1.20 µm for the distributed-load cases, indicating that the loading assumption changes the predicted response by only 4–5%, while the effect of beam non-uniformity is less than 1%.The color scale represents the deflection in micrometer.*

Supplementary Table S1: FEM results on (non) uniform beam geometries and two loading scenarios (point versus distributed)

Beam geometry	Loading	Maximum deflection (µm)
Uniform	Point load	1.25
Uniform	Distributed load	1.19
Non-uniform	Point load	1.25
Non-uniform	Distributed load	1.20

Supplementary Table S2: Two-photon polymerization printing parameters

	**Slicing** [µm]	**Hatching** [µm]	**Scan speed** [µm s^{-1}]	**Laser power** [mW]	**Contour scan speed** [µm s^{-1}]	**Contour laser power** [mW]	**Contour count**	**Contour distance** [µm]
Pedestal - base	0.3	0.3	50000	42.5	50000	42.5	12	0.2
Pedestal - micropil-lars	0.3	0.3	5000	27.5	5000	27.5	12	0.2
Support pillar	0.3	0.3	90000	40	90000	32.5	12	0.2
Beam	-	0.3	3000	38.75	-	-	-	-
Additional settings: • Interface finding was at 1.5 µm below the substrate surface. • For the printing of the beam, lines were coded in Describe with the meander setting turned on which alternates writing direction for the beam lines.								

Supplementary Table S3: Confocal imaging parameters and acquisition settings

Category	Parameter	Details
**Microscope setup**	Microscope	Nikon Ti2 inverted microscope with AX resonant scanning confocal unit
	Objective	CFI Apo LWD 40× WI Lambda DIC N2
	Numerical aperture (NA)	1.15
	Immersion medium	Water (RI = 1.333)
**Acquisition settings**	Image resolution	2048 × 2048 pixels
	Scanning mode	Resonant bidirectional scanning
	Line averaging	4×
	Zoom factor	1.00×
**Z-stack parameters**	Number of planes	95
	Step size	0.5 µm
	Total depth	~47 µm
**Laser & detection (Channel 1)**	Target	Scaffold (blue)
	Excitation	405 nm laser (0.4% power)
	Emission range	420–470 nm
**Laser & detection (Channel 2)**	Target	Actin (far red)
	Excitation	640 nm laser (1.1% power)
	Emission range	660–750 nm
**Detection settings**	Detector gain	54.6 (both channels)

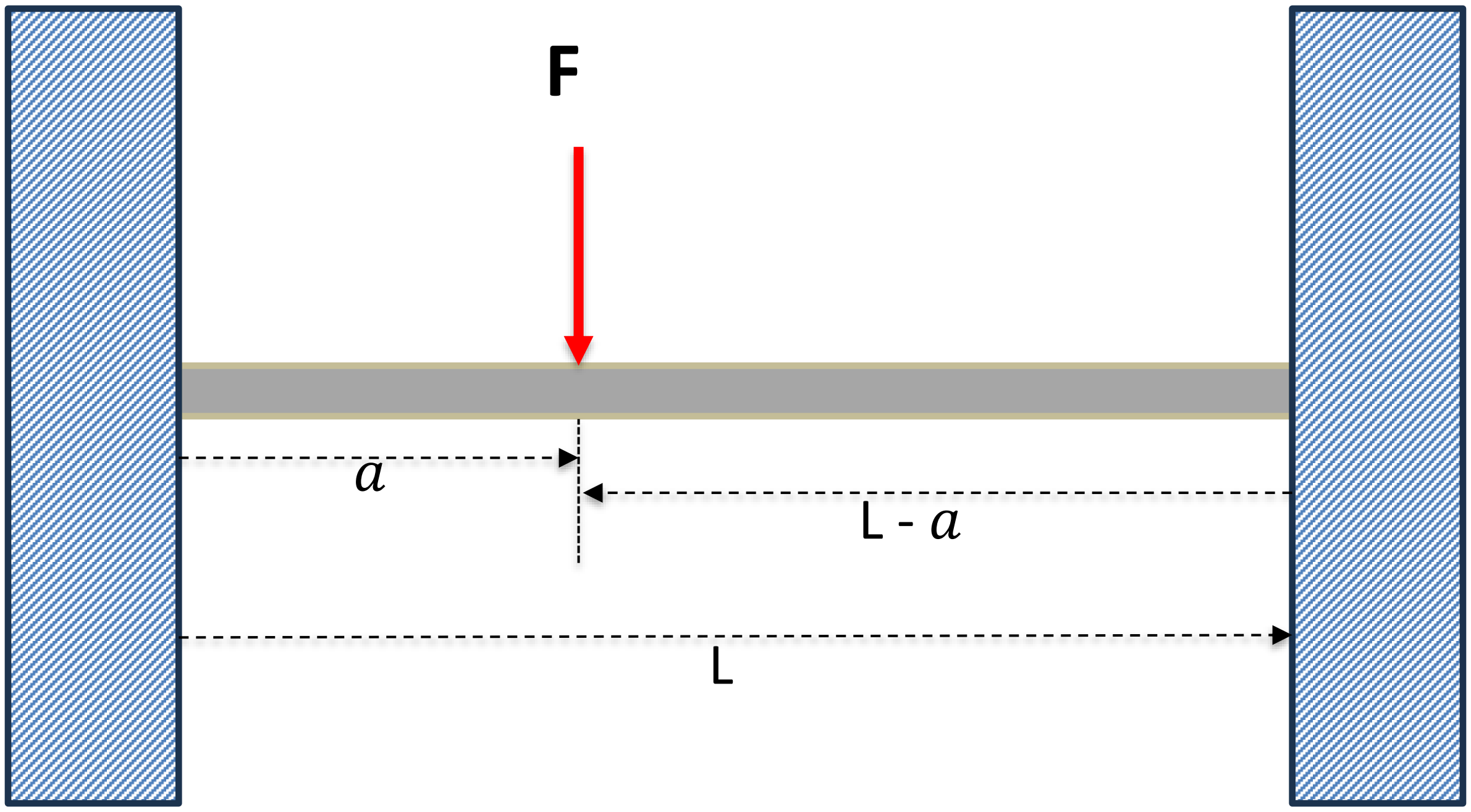

Figure S17: ***Schematic top view of a double clamped beam with a force at an arbitrary point***.

Derivation:

Beam with both ends fixed and a concentrated load at any point (see Figure S10)

The deflection at the load point is[1]:

$$\delta = \frac{Fa^3(L-a)^3}{3EI\,L^3} \qquad \text{(S1)}$$

where

a= distance from left clamping point

L= beam length

EI= flexural rigidity

Define

$$\xi = \frac{a}{L}$$

Substitute $a = \xi L \; in \; equatuon \; S1$:

$$\delta = \frac{FL^3}{3EI}\,\xi^3(1-\xi)^3$$

Check: for a center load

$$\xi = \frac{1}{2}$$

$$\delta_{mid} = \frac{FL^3}{192EI}$$

Define the mid-span stiffness

$$k_{beam} = \frac{F}{\delta_{mid}}$$

$$k_{beam} = \frac{192EI}{L^3}$$

Substitute this into the general expression:

$$\delta = \frac{F}{k_{beam}}\,64\,\xi^3(1-\xi)^3$$

Solve for F:

$$F = \frac{k_{beam}}{64\,\xi^3(1-\xi)^3}\,\delta$$

These equations were employed in MATLAB to derive the force from the deflection and the loading point

References

[1] R. J. . Roark, W. C. . Young, R. G. . Budynas, A. M. . Sadegh, *Roark's Formulas for Stress and Strain*, McGraw-Hill, **2012**.
